# Metabolomic Characterization of Sun-Dried Green Tea from Different Regions of Xishuangbanna

**DOI:** 10.3390/foods15142503

**Published:** 2026-07-15

**Authors:** Longhao Wang, Zhixia Wang, Shuai Wen, Jinhua Chen, Haibiao Chen, Jian Shen, Junjiang Lian, Dong Chen, Jianan Huang, Zhonghua Liu

**Affiliations:** 1 Key Laboratory of Tea Science of Ministry of Education, Hunan Agricultural University, Changsha 410128, China; longhaowang148@gmail.com (L.W.);; 2 Yuelushan Laboratory, Changsha 410128, China; 3 National Research Center of Engineering and Technology for Utilization of Botanical Functional Ingredients, Changsha 410128, China; 4 Yunnan Douji Tea Industry Co., Ltd., Menghai 666200, China; 5 Guyun Liuxiang Tea Co., Ltd., Kunming 650200, China; 6 Guangzhou Tea Museum, Guangzhou 510700, China; 7 Guangdong Tea Collection and Appreciation Association, Guangzhou 510091, China; 8 Key Laboratory for Evaluation and Utilization of Gene Resources of Horticultural Crops, Ministry of Agriculture and Rural Affairs of China, Hunan Agricultural University, Changsha 410128, China

**Keywords:** Sun-Dried Green Tea, flavor, metabolites, chemometrics

## Abstract

This study integrated sensory evaluation, volatile profiling (HS-SPME/GC × GC-Q-TOF-MS) and non-volatile profiling (UHPLC-Orbitrap-MS), and multivariate analysis to characterise 13 Sun-Dried Green Tea samples from Xishuangbanna, Yunnan, China. A total of 230 volatile and 226 non-volatile compounds were identified, and metabolite profiles were more similar among geographically adjacent production regions. Based on the intensity of the floral–fruity aroma, the samples were classified into three groups: strong, relatively strong, and pure, with a weak floral–fruity aroma. The strong floral–fruity group, comprising samples from Mengla and vicinity, contained high levels of Nonanal, Decanal, 2-pentylfuran, benzaldehyde, cis-linalool oxide, safranal, and 6-methyl-5-hepten-2-one. The moderately rich floral–fruity group, consisting of high-altitude Menghai samples, exhibited significantly higher levels of limonene, linalool, *p*-cymene, *β*-myrcene, *γ*-terpinene, terpinolene, and *α*-terpinene, compounds with both floral and woody attributes. Menghai and vicinity samples were characterised by higher levels of proanthocyanidins, hydrolyzable tannins, and amino acids, which account for their pronounced astringency and sweet aftertaste. In contrast, samples from Mengla and the vicinity exhibited higher levels of Myricetin 3-O-glucoside, Isoschaftoside, and Epigallocatechin 3-O-(3-O-methylgallate). Key differential compounds revealed that multiple environmental factors drive distinct metabolite profiles, which may underlie the regional quality difference of Sun-Dried Green Tea. These findings provide a theoretical reference for production improvement and market guidance.

## 1. Introduction

Pu-erh tea [*Camellia sinensis var. assamica (Mast.*)] is one of China’s most famous teas and is classified into raw Pu-erh tea and ripe Pu-erh tea based on processing technology [1]. Its production begins with Sun-Dried Green Tea, which is made from Yunnan large-leaf tea varieties within the protected geographical indication area, through sequential steps of picking, fixing, rolling, and drying. Sun-Dried Green tea, though fundamentally the raw material from which Pu-erh tea is produced, cannot be regarded merely as an in-process intermediate. It possesses unique flavor characteristics that fundamentally determine the potential for quality and economic value of Pu-erh tea [2,3]. Due to differences in altitude, climate, and soil conditions, the flavor quality of Sun-Dried Green Tea varies across regions [4]. These regional flavor variations account for the strong market recognition of specific producing areas, with Sun-Dried Green Tea from celebrated regions such as Lao Banzhang in Menghai and Yiwu in Mengla commanding a significant price premium over ordinary tea [5,6].

As Sun-Dried Green tea is the raw material for Pu-erh tea and largely determines its quality, existing research on Pu-erh tea is highly valuable for clarifying the quality of Sun-Dried Green tea [7,8]. Current studies on Pu-erh tea quality have advanced our knowledge along two dimensions: non-volatile compounds and volatile compounds. Regarding non-volatile compounds, Zhong et al. [1] found that the total content of flavan-3-ols and their derivatives, as well as phenolic acids and derivatives, in Pu-erh tea was significantly higher than in traditional green tea; simultaneously, existing studies have shown that theacrine and theobromine in Pu-erh tea exhibit a significant positive correlation with the intensity of the sweet aftertaste. In contrast, rutin, isoquercetin, and astragalin show a significant negative correlation [9]. Regarding aroma, Pang et al. [10] reported that the aroma of raw Pu-erh tea infusions is dominated by floral, sweet, and woody terpenoids (such as linalool and *α*-ionone). In contrast, the infusion of Pu-erh tea (ripe tea) is characterized by methoxyphenyl compounds (such as 1,2,3-trimethoxybenzene and 1,2,4-trimethoxybenzene) associated with aged and musty aromas. Through aroma recombination and omission experiments, *β*-ionone, *β*-damascenone, and phenylethyl alcohol were identified as key aroma contributors to the floral–fruity aroma of Pu-erh tea [11]. These studies provide scientific insights into the relationships among raw materials, processing methods, chemical composition, and flavor quality. Despite these advances, the vast majority of existing studies have focused on Pu-erh tea itself.

Xishuangbanna represents the most productive and commercially significant region for Pu-erh tea, accounting for the highest share of both total output and market sales. The final quality of tea is determined by the combined effects of processing methods, origin, and variety [12]. Among these factors, the geographical region is widely regarded as the key determinant of tea’s flavor and chemical characteristics. With respect to geographical discrimination, pentanal, heptanal, naphthalene, cedrol, and 2,6-ditert-butylbenzoquinone were identified as the key differential compounds for discriminating Pu-erh tea from the Xishuangbanna and Lincang production regions [4]. Valine, threonine, chlorogenic acid, quinic acid, epiafzelechin, and gallic acid ester were identified as key discriminatory compounds for distinguishing Pu-erh tea from the tea-growing regions of Banda, Jingmai, Nannuoshan, and Yiwu [13]. Recently, Zhou et al. [14] elucidated the metabolomic basis of regional flavor differences in Sun-Dried Green Tea across 13 regions in Yunnan Province, identifying altitude and soil available phosphorus as potential driving factors. However, the influence of chemical components on the flavor of Sun-Dried Green Tea from different production areas within the Xishuangbanna region has not been sufficiently investigated.

To address this gap, the present study comprehensively characterized Sun-Dried Green Tea samples from 13 production regions across Xishuangbanna, using sensory evaluation, HS-SPME/GC × GC-Q-TOF-MS for volatile profiling, and UHPLC-Orbitrap-MS for non-volatile metabolomics. The specific objectives were: (1) to classify samples based on sensory attributes and establish regional grouping patterns; (2) to identify key volatile and non-volatile compounds responsible for regional flavor differentiation; and (3) to explore the potential environmental drivers underlying these metabolic variations. By integrating metabolomics with multivariate analysis, this study aims to elucidate the chemical basis of regional flavor diversity in Sun-Dried Green Tea, thereby providing a theoretical foundation for quality improvement and market guidance.

## 2. Materials and Methods

### 2.1. Sample

**Figure 1:**
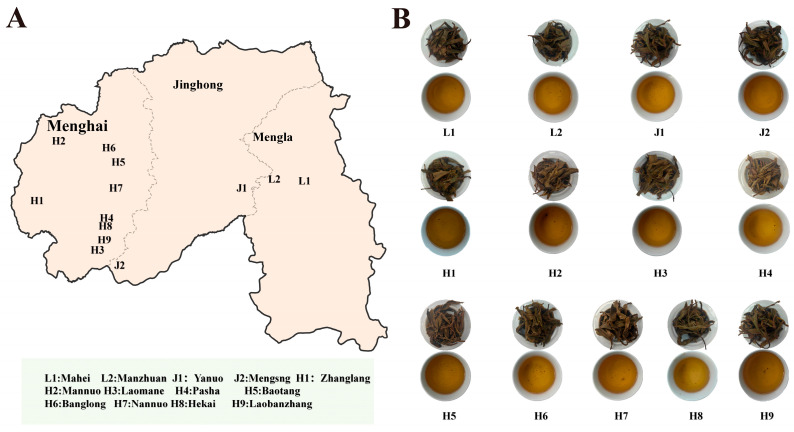
Geographical distribution of the 13 Sun-Dried Green Tea samples from Xishuangbanna, Yunnan Province, China. (**A**); The appearance and infusion of Sun-Dried Green Tea from different production regions (**B**).

13 Sun-Dried Green Tea samples were manufactured and supplied by Douji Tea Industry Company, Menghai, Yunnan, China (http://www.doujichaye.com, accessed on 25 June 2026), with fresh leaves collected from tea gardens across different regions of Xishuangbanna, Yunnan Province, China (Figure 1A). Detailed information regarding the precise geographical locations, corresponding regional names, which were named after the townships where they are located (Table 1). Samples were processed under uniform conditions in 2022, in accordance with GB/T 22111-2008 [15], to ensure consistency in processing. The tea leaves obtained were immediately sieved through a 200-mesh standard sieve to ensure a uniform particle size. After grinding, all samples were stored at 4 °C before analysis.

### 2.2. Sensory Evaluation

The sensory evaluation was conducted by five trained panellists (three men and two women, aged 23–55 years, all from Tea Science, Hunan Agricultural University), who had received at least 120 h of professional training in tea sensory evaluation. Panellists assessed each sample’s aroma and taste, providing descriptive terms for quality characteristics in accordance with the Methods of Sensory Review of Tea (GB/T 23776-2018 [16]) and the Geographical Indication Products Pu-erh tea standard (GB/T 22111-2008). After the tea infusion was poured, panellists evaluated the aroma and taste and recorded their comments. Each sample was evaluated three times on different dates.

### 2.3. HS-SPME/GC × GC-Q-TOFMS

To characterize the volatile compounds, the GC × GC-Q-TOFMS method was adapted from previous studies [17]. In brief, 0.5 g of ground tea leaves was placed in a 20 mL headspace vial. To the vial, 5 mL of boiling water and 10 μL of ethyl decanoate (10 ppm) internal standard solution are added using an automated CTC sampler. The vial was then equilibrated for 10 min at 60 °C and 600 rpm. Volatile compounds were extracted using a divinyl benzene/carboxen/polydimethylsiloxane (DVB/CAR/PDMS; 50/30 μm) fibre head (Sigma-Aldrich Trading Co., Ltd., Shanghai, China), which was inserted into the headspace vial under the same conditions for 30 min. After extraction, the SPME fibre was transferred to the gas chromatograph injector (250 °C, 10 min) for analysis. Identification of volatile compounds was performed using a GC × GC-Q-TOF-MS system equipped with HP-5MS and DB-17MS columns (Agilent Technologies, Santa Clara, CA, USA). The solid-state modulator SSM1800 (J&X Technology Co., J&X Technology Co, Shenzhen, China) was used to heat and cool between the two columns, with a modulation period of 4 s. The gas chromatograph injector temperature was maintained at 250 °C with a split ratio of 10:1. The temperature program was as follows: initial temperature of 40 °C held for 1 min, ramped at 4 °C/min to 180 °C, then increased at 20 °C/min to 250 °C, and held for 1 min.

Qualitative identification of volatile compounds was performed using Canvas software (version 1.0.0.25117), based on retention times and mass spectral matching. Retention indices (RI) were calibrated and calculated using a homologous series of n-alkanes (C7–C28). Odor descriptions of the volatile compounds were sourced from the literature and an online database (http://www.thegoodscentscompany.com/search.php, accessed on 12 May 2026). Volatile compounds were identified by comparison with NIST 20 mass spectral library data, authentic standards, and (RI). Only volatile compounds with a forward match >700, a reverse match >800, and a retention index deviation <30 retained for analysis. For quantification, the internal standard method was employed using ethyl decanoate as the reference standard. The relative response factor (f) was first calculated from a standard solution containing known amounts of the internal standard and ethyl decanoate reference, according to the following formula:

$$f\;=\;\frac{A_{s}/M_{r}}{A_{r}/M_{s}}$$

where A_s_ and A_r_ are the peak areas or peak heights of the internal standard and control, respectively, and M_s_ and M_r_ are the amounts of the internal standard and control added, respectively. Then, a sample of the component solution containing the internal standard was taken, the chromatogram recorded, and the content (M_i_) calculated from the peak response of the component solution containing the internal standard.

$$M_{i}=\frac{f\times A_{i}}{A_{s}/M_{s}}$$

where A_i_ is the peak area of the target compound, A_s_ is the peak area of the internal standard in the sample chromatogram, and M_s_ is the known amount of internal standard added to the sample. Finally, the content of each volatile compound was expressed as micrograms per kilogram of dry tea weight (μg/kg).

### 2.4. LC-MS Analysis

For the analysis of non-volatile compounds, 0.5 g of a powdered tea sample was mixed with 25 mL of a 70% (*v*/*v*) methanol–water solution and extracted via ultrasonication for 30 min. After extraction, the mixture was centrifuged at 12,000 *g* for 10 min at 10 °C. The clear supernatant was carefully collected and passed through a 0.22 μm filter before instrumental analysis. To assess the stability and repeatability of the method, pooled quality control (QC) samples were inserted into the analytical sequence.

Non-volatile compounds were measured utilizing an ultra-high-performance liquid chromatography system coupled with Orbitrap Exploris 120 mass spectrometry (UHPLC-Orbitrap-MS, Thermo Fisher Scientific, Waltham, MA, USA). The specific instrumental analysis method was performed as previously reported [18]. The chromatographic separation was performed on a UPLC system (Thermo Fisher Scientific, Waltham, MA, USA) with a mobile phase composed of water containing 0.1% formic acid (*v*/*v*) as solvent A and acetonitrile containing 0.1% formic acid as solvent B. The gradient elution program for solvent B was set as follows: 0.0–1.6 min, 0–5%; 1.6–18.0 min, 5–18%; 18.0–38.4 min, 18–95%; 38.4–42.0 min, maintained at 0%. The mass spectrometry conditions were: H-ESI ion source; spray voltages of 3500 V (positive ion mode) and 2500 V (negative ion mode); ion transfer tube temperature, 325 °C; vaporizer temperature, 300 °C; sheath gas (Arb), 35; auxiliary gas (Arb), 10; sweep gas (Arb), 1; Orbitrap resolution, 120,000; mass scan range, m/z 100–1500; scan cycle time, 3 s; RF lens amplitude, 70%. After data acquisition, qualitative identification of non-volatile compounds was performed using Compound Discoverer 3.2 (Thermo Fisher Scientific, Waltham, MA, USA) for peak picking and alignment, thereby generating a matrix of retention times and corresponding peak areas. Non-volatile compounds were annotated by combining accurate mass measurements, MS/MS fragmentation spectra, and cross-referencing with the mzVault, mzCloud, and ChemSpider. Quantitative assessment was based on the signal intensities of extracted ion features; peak integration and calibration were performed in Compound Discoverer, with the peak area of each chromatographic peak representing the relative abundance of the corresponding compound.

### 2.5. Statistical Analysis

All analyses were performed with at least three biological replicates, and data were expressed as mean ± standard deviation (SD). Statistical analysis was conducted using IBM SPSS Statistics 27(IBM, Armonk, NY, USA), with one-way analysis of variance (ANOVA) and a significance threshold of *p* < 0.05. Multivariate analyses, including principal component analysis (PCA), orthogonal partial least squares-discriminant analysis (OPLS-DA), and hierarchical cluster analysis (HCA), were performed using SIMCA-P+ 12.0 (Umetrics, Umeå, Sweden); Heatmaps were generated using the Metware Cloud, an online platform for data analysis (https://cloudmetware.cn, accessed on 25 May 2026).

## 3. Results and Analysis

### 3.1. Sensory Evaluation Result

To assess regional effects on the flavor of Sun-Dried Green Tea, a sensory evaluation was conducted on samples from 13 production regions (Table 2). Regarding aroma, samples L1, L2, and J1 exhibited the strongest floral–fruity aroma; samples H1 and H4 also possessed floral–fruity aromas, but their intensity and persistence were notably lower than those of the first three; the remaining samples were characterized primarily by a weak floral–fruity aroma. In terms of taste, samples L1, L2, and J1 were primarily characterized as mellow. The remaining samples exhibited a heavy, mellow, and thick profile accompanied by astringency, with H3, H4, H8, and H9 additionally displaying a sweet aftertaste. Geographically, samples L1, L2, and J1 were concentrated in the same region (Mengla and vicinity). In contrast, the remaining samples were distributed in another relatively distant but adjacent region (Menghai and vicinity). The regional differences in the aforementioned sensory qualities suggest that the composition and content of chemical constituents may be systematically dependent on origin. This finding is consistent with existing research indicating that teas from similar production regions often share quality characteristics [19]. Geographical origin may be a pivotal factor in the flavor variations of Sun-Dried Green Tea.

### 3.2. Identification of Volatile and Non-Volatile Metabolites and Sample Grouping by Production Region

HS-SPME/GC × GC-Q-TOF-MS further characterized the volatile compounds of Sun-Dried Green Tea. A total of 230 volatile compounds were identified across the 13 Sun-Dried Green Tea samples, including 40 olefins, 38 aromatic hydrocarbons, 32 alcohols, 29 ketones, 26 aldehydes, 25 alkanes, 18 esters, 16 heterocyclic compounds, 3 phenols, and 3 ethers. In the 13 Sun-Dried Green Tea samples, the proportions of volatile constituents were as follows: alcohols (29.00–35.13%), olefins (27.58–34.03%), aldehydes (8.95–15.11%), heterocyclic compounds (6.57–9.20%), ketones (5.75–9.92%), aromatic hydrocarbons (3.75–5.44%), ethers (2.69–4.74%), alkanes (0.96–1.95%), and phenols (0.05–0.17%). The combined proportion of alcohols, olefins, and aldehydes exceeded 70% of the total volatile content, suggesting that these classes of compounds contribute substantially to the aroma formation in Sun-Dried Green Tea. Total volatile content across all samples ranged from 4673.64 ± 95.15 μg/kg to 7086.66 ± 14.03 μg/kg. Notably, Sun-Dried Green Tea from region H1 exhibited the highest total volatile content, which was significantly higher than that of samples from other regions. In contrast, samples from region H3 displayed the lowest total volatile content. Among all samples, the alcohol content ranged from 1468.40 to 2370.37 μg/kg. Region H1 samples showed the highest alcohol content, significantly exceeding those of the other regions, while region H6 samples exhibited the lowest. Similarly, the olefin content ranged from 1426.84 to 2276.99 μg/kg, with the highest concentration observed in region H1 and the lowest in region H3. The number of detected volatile compounds in the 13 Sun-Dried Green Tea samples ranged from 128 to 151, of which a total of 88 were common to all production regions. Common compounds accounted for 55% to 65% of the total number of volatile compounds. Still, they constituted more than 85% of the total volatile compound mass, forming the core aroma profile of Sun-Dried Green Tea. In addition, several volatile compounds with characteristic odor profiles exhibited region-specific distributions: Borneol (woody) and Butyl butanoate (fruity, sweet) were detected only in L2; methyl benzoate (floral–fruity) was found only in J1; and ethyl (*Z*)-hex-3-enoate (floral, fruity) and 2,5-dimethylcyclohexanol (woody) were detected only in H2.

**Figure 2:**
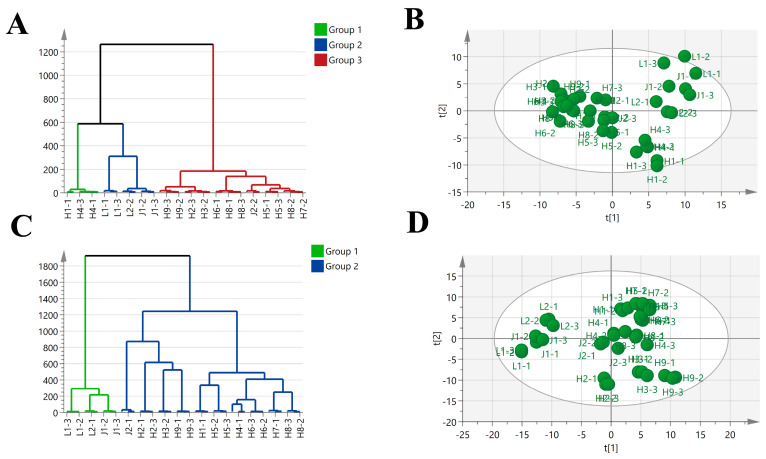
HCA for volatile compounds (**A**). Score plot of PCA for volatile compounds (**B**). HCA for non-volatile compounds (**C**). Score plot of PCA for non-volatile compounds (**D**).

To investigate the commonalities and differences in volatile compounds contributing to the aroma formation of Sun-Dried Green Tea from different production regions, hierarchical cluster analysis (HCA) was performed. The HCA results showed that the 13 samples were classified into three distinct clusters (Figure 2A). Based on the HCA clustering pattern, together with the aroma characteristics obtained from sensory evaluation, the samples were divided into three groups: Group 1 (L1, L2, J1), Group 2 (H1, H4), and Group 3 (J2, H2, H3, H5, H6, H7, H8, H9). Both sensory attributes and geographical distribution strongly supported this grouping. Group 1 samples, all originating from Mengla and vicinity, exhibited the Strongest floral–fruity aroma. Group 2 samples from Menghai also exhibited a floral–fruity aroma, but with notably lower intensity and persistence than those of Group 1. Group 3 samples, also from Menghai and vicinity, geographically proximate to Group 2, exhibited a pure aroma with only a weak floral–fruity aroma. The consistency among the HCA dendrogram, sensory evaluation, and production regions validates the rationality of this three-group classification. It provides a reliable basis for the subsequent differential analysis of volatile compounds. Consistent with the HCA results, the PCA score plot revealed a clear separation pattern corresponding to the grouping (Figure 2B). Samples L1, L2, and J1 were clustered in the first quadrant. In contrast, samples H1 and H4 were distributed in the fourth quadrant. The remaining samples were separated from these five samples along PC1, primarily located in the second and third quadrants, with a relatively compact internal distribution.

The non-volatile compounds in Sun-Dried Green Tea were comprehensively characterized via UHPLC-Orbitrap-MS-based untargeted metabolomics of samples from 13 production regions. Based on retention time, mass-to-charge ratio, molecular weight, and adduct ions, and MS/MS fragmentation spectra by matching against our in-house library and public databases, 226 non-volatile compounds were identified [18,20]. These were classified into 11 categories: flavonoids (65), catechins and their derivatives (28), phenolic acids (25), amino acids and their derivatives (23), organic acids (23), phenols (13), nucleotides and their derivatives (14), sugars (9), alkaloids (5), lipids (10), and others (11).

Similarly, HCA was performed on the 226 non-volatile compounds. HCA showed that samples L1, L2, and J1 clustered together. In contrast, the remaining samples formed another cluster (Figure 2C). This result was consistent with the sensory evaluation, which indicated that, except for L1, L2, and J1, the other samples exhibited pronounced astringency. Therefore, for the subsequent non-volatile analysis, the 13 samples were divided into two groups: Group 1 (Mengla and vicinity, 3 samples) and Group 2 (Menghai and vicinity, 10 samples). Similar to the HCA results, PCA also showed that L1, L2, and J1 clustered together, and the remaining samples were separated from these three along PC1 (Figure 2D). The discrepancy between the clustering results of volatile and non-volatile metabolites may be attributed to their differential sensitivity to environmental factors [21].

### 3.3. Analysis of Volatile Metabolites in Sun-Dried Green Tea from Different Production Regions

**Figure 3:**
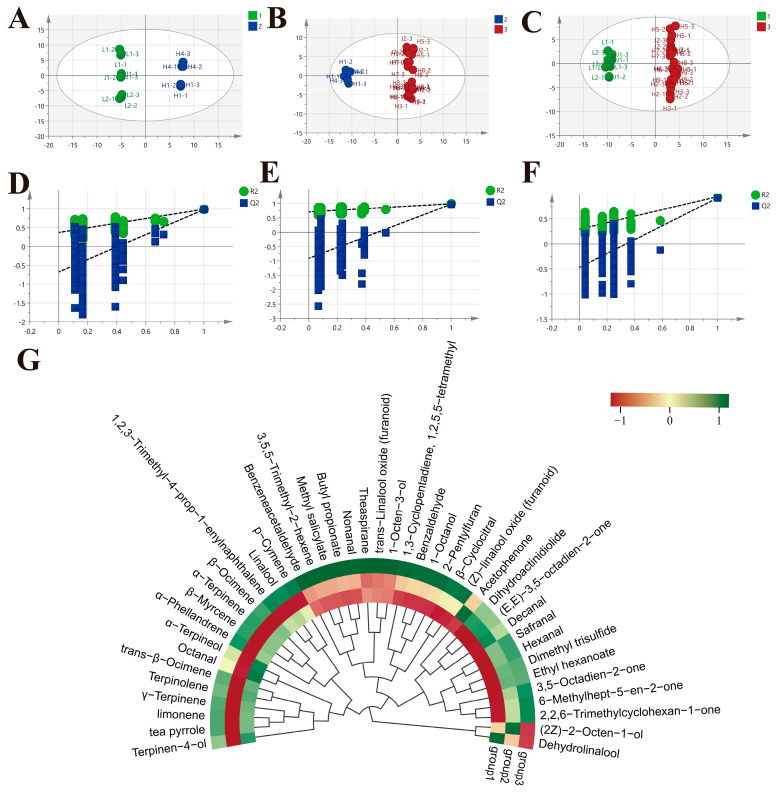
OPLS-DA analysis of volatile compounds and heatmap of key differential volatile compounds among three groups. Score plots of OPLS-DA for volatile compounds: Group 1 vs. Group 2 (**A**), Group 1 vs. Group 3 (**B**), Group 2 vs. Group 3 (**C**); Cross-validation results: Group 1 vs. Group 2 (**D**), Group 1 vs. Group 3 (**E**), Group 2 vs. Group 3 (**F**); Heatmap of key differential volatile compounds among the three groups (**G**).

To further identify the key aroma compounds differentiating the three groups of Sun-Dried Green Tea, OPLS-DA was performed between each pair of the three groups. The three OPLS-DA models exhibited strong explanatory and predictive abilities: Group 1 vs. Group 2 (R^2^X = 0.72, R^2^Y = 0.99, Q^2^ = 0.98), Group 1 vs. Group 3 (R^2^X = 0.71, R^2^Y = 0.99, Q^2^ = 0.97), and Group 2 vs. Group 3 (R^2^X = 0.69, R^2^Y = 0.98, Q^2^ = 0.92). The 200 permutation tests confirmed no overfitting for all three models (R^2^ = 0.36, Q^2^ = −0.71; R^2^ = 0.70, Q^2^ = −0.97; and R^2^ = 0.29, Q^2^ = −0.52, respectively) (Figure 3A–F). Metabolites were screened using the combined criteria of VIP > 1, fold change (FC) > 1.2 or < 0.833, and false discovery rate (FDR)-adjusted *p* < 0.05 [22]. Based on this screening, 43 compounds were identified as potential key differential volatile compounds associated with the different aroma characteristics among the three groups. These compounds contributed substantially to the differentiation of aroma among the groups. Specifically, 14 key differential volatile compounds were consistently identified across all three pairwise OPLS-DA models, namely limonene (fruity, floral, woody), linalool (floral, sweet, woody), *α*-terpineol (woody), terpinolene (herbal, woody), tea pyrrole (burnt, roasted), *p*-cymene (herbal, fruity), γ-terpinene (woody), *α*-terpinene (woody), theaspirane (herbal, sweet), benzeneacetaldehyde (green, floral), 2-pentylfuran (fruity, green), benzaldehyde (sweet, woody), and dehydrolinalool (floral, woody) [23,24]. With the exception of dehydrolinalool, which was significantly more abundant in Group 3 than in Groups 1 and 2, all of these compounds were present at significantly higher levels in Groups 1 and 2, both of which exhibited stronger floral–fruity aroma characteristics than Group 3. A total of 27 key differential volatile compounds were identified between Groups 1 and 2. The compounds enriched in Group 1 were predominantly lipid oxidation-derived volatiles, including nonanal (floral, fresh), decanal (sweet, floral), and (E,E)-3,5-octadien-2-one (fruity, green); carotenoid-derived compounds, such as safranal (herbal) and 6-methylhept-5-en-2-one (fruity, green); and esters, represented by ethyl hexanoate (sweet, fruity). By contrast, the compounds enriched in Group 2 were mainly terpenoids, including limonene, linalool, *α*-terpineol, terpinolene, *p*-cymene, *γ*-terpinene, α-terpinene, *β*-myrcene (fruity), *β*-ocimene (woody, floral), trans-*β*-ocimene (sweet, herbal), and terpinen-4-ol (woody), along with tea pyrrole, a pyrrole aldehyde typically generated via the Maillard reaction or Strecker degradation during thermal processing [3,7,23,25].

Subsequently, 26 key differential volatile compounds were identified based on the combined criteria of VIP > 1, FDR-adjusted *p* < 0.05, to investigate regional metabolite variation across the 13 production regions. The model parameters (R^2^X = 0.94, R^2^Y = 0.94, Q^2^ = 0.78) indicated good explanatory and predictive ability, and the 200-permutation test confirmed no overfitting (R^2^ = 0.52, Q^2^ = −0.69) (Figure S1). Furthermore, these compounds showed a high degree of similarity with the key differential volatile compounds identified among the three aroma groups of Sun-Dried Green Tea. Among these, terpenoids and their oxidation products (e.g., limonene, linalool, linalool oxide, and hotrienol), together with carotenoid-derived compounds (e.g., safranal, *β*-cyclocitral, and *β*-ionone), were the predominant contributors, accounting for the observed variation [22,24].

### 3.4. Analysis of Non-Volatile Metabolites in Sun-Dried Green Tea from Different Production Regions

**Figure 4:**
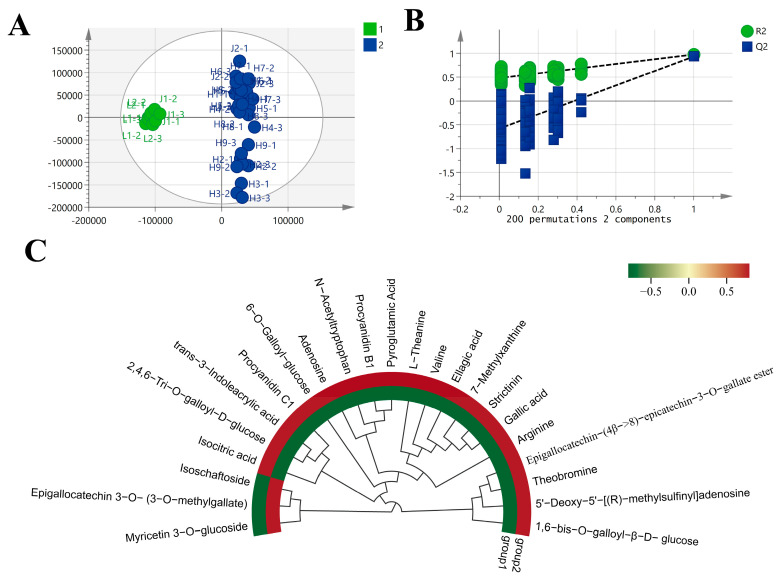
Score plot of the OPLS-DA for non-volatile compounds (**A**). Cross-validation results of the OPLS-DA for non-volatile compounds (**B**). Heatmap of key differential non-volatile compounds for Sun-Dried Green Tea between two groups (**C**).

OPLS-DA model was subsequently constructed to analyse the differences in non-volatile compounds between the two groups. The model parameters (R^2^X = 0.84, R^2^Y = 0.98, Q^2^ = 0.97) indicated good explanatory and predictive ability (Figure 4A). After 200 permutation tests, the results showed no overfitting (R^2^ = 0.30, Q^2^ = −0.78) (Figure 4B). The results demonstrated significant differences in the non-volatile compounds of Sun-Dried Green Tea from the two groups. Based on the combined criteria of VIP > 1, FC > 1.2 or < 0.833, and FDR-adjusted *p* < 0.05, a total of 23 key differential non-volatile compounds were identified between the two groups, including 5 amino acids and their derivatives, 4 phenols, 4 catechins and their derivatives, 2 flavonoids, 2 phenolic acids, 2 alkaloids, 2 nucleotides and their derivatives, and 2 organic acids. These non-volatile compounds played important roles in both the formation of taste and the differentiation among groups. Subsequently, a heatmap was used to visualise variation in the content of these 23 differential compounds (Figure 4C). Only three non-volatile compounds, namely epigallocatechin 3-O-(3-O-methylgallate), myricetin 3-O-glucoside, and isoschaftoside, were significantly more abundant in Group 1 than in Group 2. Epigallocatechin 3-O-(3-O-methylgallate) is an o-methylated epigallocatechin gallate naturally present in tea [26]. Previous studies have shown that epigallocatechin 3-O-(3-O-methylgallate) is a key compound for distinguishing teas from different geographical origins [27]. This is in marked contrast to the volatile compounds, which were enriched in the L1, L2, and J1 samples of Group 1, highlighting a distinct divergence between volatile and non-volatile metabolite accumulation in this region.

Subsequently, key differential non-volatile compounds (VIP > 1, FDR-adjusted *p* < 0.05) were identified to investigate regional metabolite variation across the 13 production regions. The model exhibited good explanatory and predictive performance (R^2^X = 0.98, R^2^Y = 0.96, Q^2^ = 0.86) without overfitting, as validated by the 200 permutation test (R^2^ = 0.45, Q^2^ = −0.75) (Figure S2). 29 key differential non-volatile compounds were screened, with catechins and their derivatives constituting the largest class, followed by flavonoids, phenols, and amino acids, which collectively drove the differentiation between groups. This finding is consistent with previous studies demonstrating that the composition and content of catechins can serve as chemical fingerprints for distinguishing teas from different production regions [28].

## 4. Discussion

Variations in temperature, altitude, precipitation, and other environmental factors influence the quality of tea produced in different regions. In this study, sensory evaluation revealed a clear dichotomy in the flavor quality of Sun-Dried Green Tea samples from Xishuangbanna: samples from Mengla and the vicinity exhibited a stronger floral–fruity aroma and a mellow taste, whereas samples from Menghai and the vicinity were characterised by astringency and a weaker floral–fruity aroma. Through integrated analyses of volatile and non-volatile metabolomics, we identified the key chemical compounds driving these regional differences.

### 4.1. Volatile Compounds Contributing to Regional Differences in Aroma of Sun-Dried Green Tea

Similar to Pu-erh tea, Sun-Dried Green Tea made from Yunnan large-leaf varieties has a pure, floral-fruit aroma as its primary flavor characteristics. In agreement with previous reports, limonene, *β*-ionone, linalool, *β*-myrcene, benzaldehyde, theaspirane, *α*-pinene, and *α*-terpineol, which are recognized as key aroma-active compounds in Pu-erh tea, were consistently detected across all samples and constituted the core aroma of Sun-Dried Green Tea [3,11,29].

Despite having the strongest floral–fruity aroma, Group 1 displayed a lower total volatile content (6123.24 ± 245.28 μg/kg) compared with Group 2 (6784.53 ± 302.19 μg/kg), while still exceeding that of Group 3 (5230.57 ± 298.81 μg/kg). This suggests that aroma quality is determined not solely by the total quantity of volatile compounds, but rather by the composition and relative proportions. Comparison of the key differential volatile compounds among the three groups revealed that terpenoids, including limonene, linalool, and *p*-cymene, as well as certain aromatic aldehydes such as benzeneacetaldehyde and benzaldehyde, were present at significantly higher levels in Groups 1 and 2 than in Group 3. These compounds were the primary contributors to the pronounced floral–fruity aroma observed in Groups 1 and 2. Although Group 2 exhibited significantly higher levels of floral terpenoids than Group 1, a range of woody terpenoids, including γ-terpinene, *α*-terpinene, *α*-terpineol, and terpinolene, along with 2-pentylfuran (green) and tea pyrrole (roasted), were also significantly enriched in Group 2 relative to Group 1. Meanwhile, the volatile compounds enriched in Group 1 have been widely reported to contribute to the enhancement of floral–fruity aroma and overall aroma quality. Previous studies have confirmed that safranal, 6-methyl-5-hepten-2-one, and 2-pentylfuran are key contributors to the floral–fruity aroma of Yunnan Sun-Dried Green Tea, and that their masking effect on roasted aromas ultimately renders the floral–fruity notes more perceptible [7]. Decanal, a fatty aldehyde generated from oleic acid via the lipoxygenase and hydroperoxide lyase pathway during tea processing, has been identified as a key compound enriched in tea trichomes that plays an important role in enhancing the floral–fruity aroma of white tea [30]. Although floral terpenoids were enriched in Group 2, the co-occurrence of woody, green, and roasted volatile compounds may lead to perceptual interactions within the tea infusion matrix, partially attenuating the perception of pure floral–fruity aroma [31,32]. For example, the competitive binding of *α*-terpineol and floral volatiles to olfactory receptors such as OR1A1 and OR1D2 may reduce the perceived intensity of floral aroma [33]. We therefore speculate that the regional aroma quality of Sun-Dried Green Tea is governed not by the absolute abundance of individual floral compounds, but by the compositional proportions among multiple volatile compounds that collectively shape the overall aroma presentation.

### 4.2. Non-Volatile Compounds Contributing to Regional Differences in Taste of Sun-Dried Green Tea

Regarding non-volatile compounds, OPLS-DA analysis identified 23 key differential compounds that clearly distinguished the two groups of samples. These two groups of taste-differentiating compounds are primarily responsible for the observed differences in taste between regions.

The levels of proanthocyanidins and hydrolyzable tannins were significantly higher in Group 2 samples than in Group 1. The catechins and their derivatives identified in this study comprised two proanthocyanidins (procyanidin B1 and procyanidin C1) and two catechin derivatives (epigallocatechin 3-O-(3-O-methylgallate) and epigallocatechin-(4*β* → 8)-epicatechin-3-O-gallate ester). In this study, the level of Epigallocatechin 3-O-(3-O-methylgallate) was significantly higher in Group 1 than in Group 2, whereas the remaining catechin derivatives were significantly higher in Group 2 than in Group 1. These compound classes are well-established as contributors to bitterness and astringency in tea infusions [34]. Among them, proanthocyanidins, characterized by their polyphenolic structures, exhibit intense astringency and may be the primary cause of the pronounced astringency in group 2 samples [35]. This is consistent with previous studies identifying catechins and their derivatives as the principal determinants of the bitter-astringent character of Pu-erh tea [36].

Organic acids are organic compounds containing one or more carboxyl functional groups, while phenolic acids are organic acids that contain one or more phenolic groups [37]. Although present at relatively low concentrations, they play a crucial role in enhancing the complexity of tea infusions and modulating sensory balance [38]. In this study, the levels of gallic acid, Neochlorogenic acid, trans-3-Indoleacrylic acid, ellagic acid, and isocitric acid were significantly higher in Group 2 than in Group 1. Among the various families of phenolic compounds, tannins are recognised as the primary compounds associated with astringency in foods. Notably, four hydrolyzable tannins (strictinin, 2,4,6-tri-O-galloyl-D-glucose, 6-O-galloyl-glucose, and 1,6-bis-O-galloyl-*β*-D-glucose) were previously reported to exhibit a strong correlation with tea grade and sweet aftertaste [39]. It was hypothesized that these gallotannins may undergo partial hydrolysis in the oral environment, releasing free glucose and gallic acid, thereby contributing to a sweet aftertaste [35,39]. Sweet aftertaste is a psychological perception in which sweetness is relatively amplified following the subsidence of astringency, resulting from the slow release of sweet-tasting compounds from salivary protein complexes and the release of free sugars via enzymatic hydrolysis by saliva [35,40]. Previous studies have indicated that the sweet aftertaste of Pu-erh tea is associated with the concentrations of theophylline, EGCG, theobromine, theanine, and gallic acid, as well as their interactions [39]. Although H3, H4, H8, and H9 exhibited a sweet aftertaste in Group 2, which was characterized by strong astringency and also had high levels of these compounds, the specific mechanism still needs further investigation. In addition, amino acids, including L-theanine, L-valine, and arginine, were significantly higher in group 2. At appropriate concentrations, these amino acids can alleviate the sharp, monotonous astringency imparted by caffeine and catechins, thereby enhancing the tea infusion’s taste complexity [35,41]. From the perspective of the key differential non-volatile compounds, the balanced interplay among multiple classes of compounds played a crucial role in shaping the taste profile of the tea infusion, which also explains why the sensory evaluation revealed prominent mellow thickness and astringency without pronounced bitterness. The synergistic effects of multiple classes of non-volatile compounds collectively shape the characteristic taste layers of Sun-Dried Green Tea, including mellow thickness, astringency, and sweet aftertaste [42,43].

### 4.3. Key Differential Metabolites Across Production Regions and Their Driving Factors

The key differential metabolites distinguishing production regions overlapped considerably with those responsible for regional differences in flavor quality. A clear geographical trend was observed, whereby samples from adjacent regions tended to cluster together based on their metabolite profiles. Moreover, characteristic Pu-erh tea metabolites such as theacrine and strictinin were identified among the key differential compounds with high VIP scores, suggesting that the chemical foundation for the distinct quality attributes of Pu-erh tea is already established in the Sun-Dried Green Tea stage [44].

Samples from Mengla and its vicinity (L1, 1330 m; L2, 1300 m; J1, 1400 m) generally contained higher levels of monoterpenoids, including linalool, limonene, trans-linalool oxide (furanoid), and *p*-cymene. A similar trend was observed in H1 (2050 m) and H4 (1800 m), two samples from the Menghai that were collected at the highest altitudes among all samples. In contrast, the Mengla and vicinity samples were characterized by elevated levels of carotenoid-derived compounds (e.g., 2,2,6-trimethylcyclohexanone and safranal) and lipid-oxidation-derived volatiles (e.g., 1-octen-3-ol and 2-pentylfuran). A previous study comparing raw Pu-erh tea from different regions of Yunnan reported that tea plants in Mengla are characterized by significantly higher levels of terpenoids and retained higher lipoxygenase pathway activity during both the fresh leaf stage and primary processing, which contributed to their more pronounced floral–fruity aroma [45]. This finding is consistent with the results of the present study. However, the precise mechanisms underlying the observed regional differences remain to be fully elucidated.

Regarding non-volatile compounds, samples from Menghai and the vicinity were generally characterised by higher concentrations of catechins and their derivatives, flavonoids, phenolic compounds, alkaloids, and amino acids. Among these, the high-altitude Menghai samples H1 (2050 m) and H4 (1800 m) stood out for their particularly elevated levels of amino acids, notably L-theanine and valine, as well as the organic acid trans-3-indoleacrylic acid. From an environmental perspective, compared with Mengla, Menghai features high-altitude mountainous terrain, intense sunlight, strong UV radiation, relatively low precipitation, and a pronounced large diurnal temperature range. The altitude of the Menghai samples in the present study ranged from 1400 to 2050 m. Strong UV radiation is a potent elicitor of the phenylpropanoid pathway, directly upregulating key genes such as PAL and CHS, thereby channelling carbon flux toward the biosynthesis of bitterness- and astringency-related compounds, including phenolic acids, catechins, and flavonoids [46,47]. Specifically, intense sunlight can activate photoreceptors and downstream transcription factors, including CsGLK and R2R3-MYB, thereby upregulating the expression of key enzyme genes involved in catechin biosynthesis [48]. Strong UV radiation, by inducing oxidative stress, further stimulates tea plants to accumulate phenolic compounds such as catechins, which act as photoprotective agents that scavenge reactive oxygen species [49,50]. We therefore speculate that this climatic contrast is responsible for the enrichment of non-volatile bitter-astringent compounds in Menghai and its vicinity.

Altitude-associated changes in light and temperature further contribute to metabolic heterogeneity within Menghai and its vicinity. The diffuse light conditions prevalent at higher altitudes promote nitrogen metabolism in tea plants, thereby facilitating the biosynthesis and accumulation of amino acids, organic acids, and phenolic acids [51,52]. This is in line with the enrichment of both non-volatile and volatile compounds observed in samples H1 (2050 m) and H4 (1800 m). Furthermore, variations in light intensity and quality induced by altitude have been shown to critically shape volatile compounds accumulation in tea plants, providing a plausible mechanistic explanation for the relatively prominent floral–fruity aroma characteristics of H1 and H4 within the Menghai and vicinity [53]. It is noteworthy, however, that altitude alone did not appear to be the dominant driver of the inter-regional divergence between Menghai and Mengla, given that the lower-elevation Mengla samples (L1, L2, and J1; 1300–1400 m) exhibited a more pronounced floral–fruity aroma. A recent study on the quality characteristics of Sun-Dried Green Tea from different regions of Yunnan similarly emphasized the integrated roles of altitude and soil mineral elements in shaping tea metabolite profiles [14]. Although the present study focused on the Xishuangbanna region and many of the identified differential metabolites are also influenced by soil mineral composition, the inter-regional metabolic differentiation is more likely governed by the combined effects of multiple environmental factors. Future studies are therefore warranted to explore how light quality, temperature, humidity, soil minerals, and microbial communities collectively interact with tea metabolism to shape the regional quality of Sun-Dried Green Tea.

It should be noted that the sample set used in this study, while representative of the well-known production regions within Xishuangbanna, was limited in size and scope. Additionally, environmental factors such as light intensity and temperature were inferred from general climatic descriptions rather than directly measured at each sampling site. Nevertheless, the integration of sensory evaluation with comprehensive metabolomic analysis has provided valuable insights into the regional flavor differentiation of Sun-Dried Green Tea.

## 5. Conclusions

In this study, we found that Sun-Dried Green Tea from different producing regions in Xishuangbanna, Yunnan, China, exhibits distinct regional characteristics in sensory quality. By analysing their non-volatile and volatile metabolite profiles with multivariate statistical analysis, we further examined differences in flavor quality among Sun-Dried Green Tea samples from various regions. A total of 230 volatile compounds were detected using GC × GC-Q-TOF-MS. Floral terpenoids, including linalool and limonene, showed significant differences among the samples, with the highest levels in the high-altitude Menghai samples, followed by the Mengla samples, and the lowest in the remaining Menghai samples. In the high-altitude Menghai samples, the concomitant enrichment of woody terpenoids, such as γ-terpinene, terpinolene, and *α*-terpinene, may have partially attenuated the perception of pure floral notes. By contrast, the Mengla samples were distinguished by elevated levels of lipid oxidation-derived volatiles, including nonanal and decanal, and carotenoid-derived compounds, such as 6-methyl-5-hepten-2-one, which likely contributed to their most pronounced floral–fruity aroma. A total of 226 non-volatile compounds were identified using UHPLC-Orbitrap-MS. Comparative analysis of Sun-Dried Green Tea samples with contrasting taste profiles revealed that the combined effects of catechins and their derivatives and hydrolyzable tannins may be the primary contributors to the pronounced astringency and mellow thickness characteristic of the Menghai samples. Environmental factors, including light quality, temperature, and altitude, were identified as the primary drivers of the sensory quality divergence of Sun-Dried Green Tea across production regions, with their effects being particularly pronounced within the Menghai area. Future research should further explore the interactions between environmental factors, such as soil mineral elements, and tea metabolites to deepen our understanding of the mechanisms underlying the quality formation of Sun-Dried Green Tea across different production regions.

## Figures and Tables

**Table 1:** Basic information of 13 Sun-Dried Green Teas from different regions.

No.	Location	Name	Altitude (m)
1	Mahei Village, Yiwu Town, Mengla County, Xishuangbanna, Yunnan Province, China	L1	1330
2	Manzhuan Village, Xiangming Township, Mengla County, Xishuangbanna, Yunnan Province, China	L2	1300
3	Yanuo Village, Jinuo Ethnic Township, Jinghong City, Xishuangbanna, Yunnan Province, China	J1	1400
4	Mengsong Village, Damenglong Town, Jinghong City, Xishuangbanna, Yunnan Province, China	J2	1600
5	Zhanglang Village, Xiding Township, Menghai County, Xishuangbanna, Yunnan Province, China	H1	2050
6	Mannuo Village, Mengwang Township, Menghai County, Xishuangbanna, Yunnan Province, China	H2	1400
7	Laoman’e Village, Bulangshan Township, Menghai County, Xishuangbanna, Yunnan Province, China	H3	1580
8	Pasha Village, Gelanghe Hani Ethnic Township, Menghai County, Xishuangbanna, Yunnan Province, China	H4	1800
9	Baotang Village, Mengsong Township, Menghai County, Xishuangbanna, Yunnan Province, China	H5	1700
10	Banglong Village, Mengsong Township, Menghai County, Xishuangbanna, Yunnan Province, China	H6	1610
11	Nannuo Village, Gelanghe Township, Menghai County, Xishuangbanna, Yunnan Province, China	H7	1600
12	Hekai Village, Menghun Town, Menghai County, Xishuangbanna, Yunnan Province, China	H8	1500
13	Laobanzhang Village, Bulangshan Township, Menghai County, Xishuangbanna, Yunnan Province, China	H9	1670

**Table 2:** Sensory evaluation results.

No.	Aroma	Taste
L1	Strong floral–fruity aroma	Mellow and thick
L2	Strong floral–fruity aroma	Mellow
J1	Strong floral–fruity aroma	Mellow and thick
J2	Weak fruity aroma	Heavy and thick, astringent
H1	Relatively Strong floral–fruity aroma	Relatively mellow, astringent
H2	Weak floral–fruity aroma	Mellow and thick, astringent
H3	Weak floral–fruity aroma	Mellow, and astringent with a sweet aftertaste
H4	Relatively Strong floral–fruity aroma	Mellow, and astringent with a sweet aftertaste
H5	Weak fruity aroma	Relatively heavy and thick, astringent
H6	Weak floral–fruity aroma	Relatively mellow, astringent
H7	Lasting pure aroma	Mellow and thick, astringent
H8	Pure aroma with weak fruity aroma	Relatively mellow and thick, astringent with a sweet aftertaste
H9	Lasting pure aroma with weak floral aroma	Heavy and thick, astringent with a sweet aftertaste

## Data Availability

The original contributions presented in this study are included in the article/Supplementary Materials. Further inquiries can be directed to the corresponding authors.

## Supplementary Materials

The following supporting information can be downloaded at: https://www.mdpi.com/article/10.3390/foods15142503/s1. Figure S1: Key differential volatile compounds distinguishing Sun-Dried Green Tea from different production regions. OPLS-DA score plot of volatile compounds (A). Cross-validation results of the OPLS-DA models (B). Heatmap of key differential volatile compounds (C). Figure S2: Key differential non-volatile compounds distinguishing Sun-Dried Green Tea from different production regions. OPLS-DA score plot of non-volatile compounds (A). Cross-validation results of the OPLS-DA models (B). Heatmap of key differential non-volatile compounds (C). Table S1: Identification of volatile compounds in Sun-Dried Green Tea. Table S2: Identification of non-volatile compounds in Sun-Dried Green Tea.
