# Supplementary material for: Metabolomic Characterization of Sun-Dried Green Tea from Different Regions of Xishuangbanna
Source: Foods. 2026 Jul 15;15(14):2503. doi: 10.3390/foods15142503 (PMC13407954; doi:10.3390/foods15142503)
Supplement: Supplementary file 1 [file foods-15-02503-s001.zip › foods-4380069-Supplementary Materials.pdf]

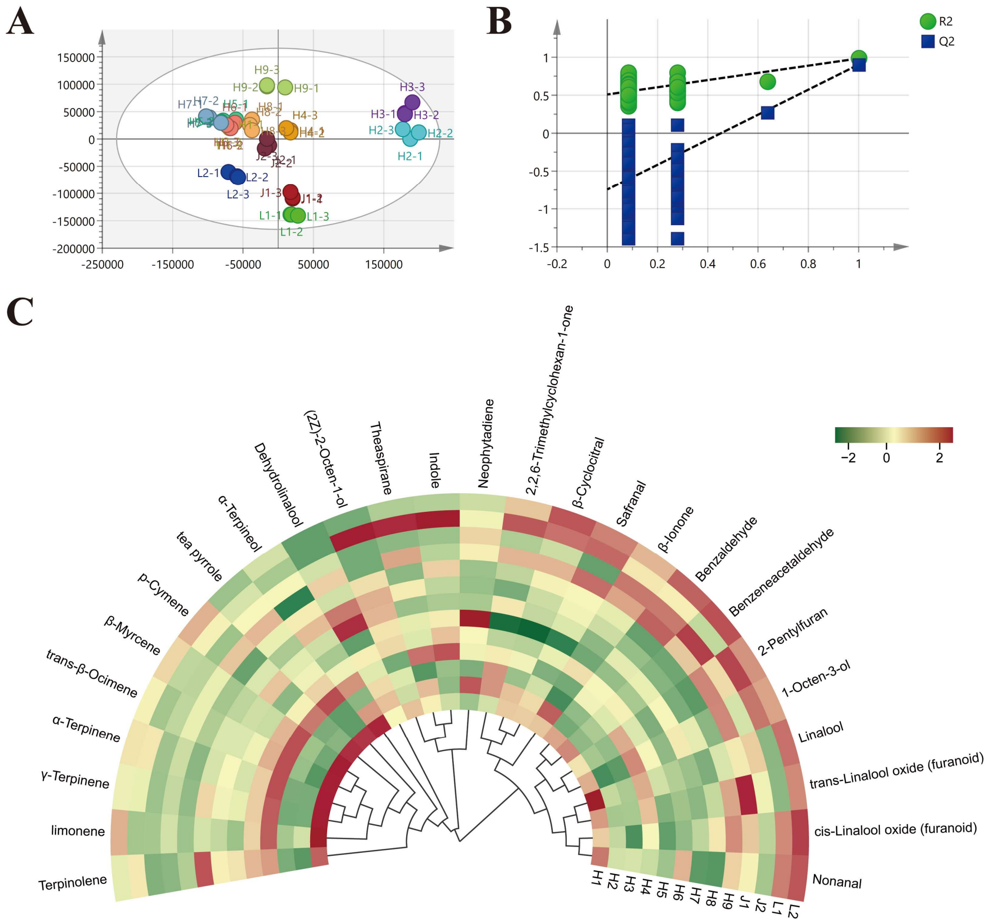

Figure S1. key differential volatile compounds distinguishing Sun-Dried Green Tea from different production regions. OPLS-DA score plot of volatile compounds (A). Cross-validation results of the OPLS-DA models (B). Heatmap of key differential volatile compounds (C).

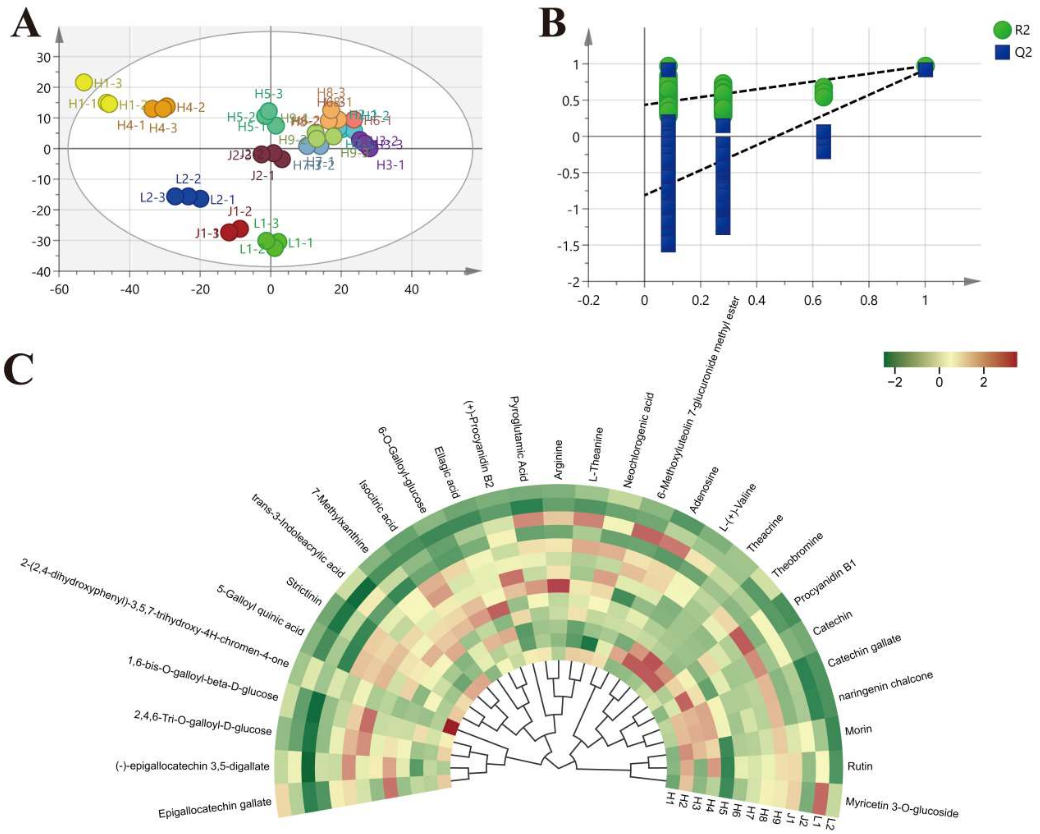

Figure S2. key differential non-volatile compounds distinguishing Sun-Dried Green Tea from different production regions. OPLS-DA score plot of non-volatile compounds (A). Cross-validation results of the OPLS-DA models (B). Heatmap of key differential non-volatile compounds (C).

Table S1. Identification of volatile compounds in Sun-Dried Green Tea

NO	CAS	Volatile component	Concentration (μg/kg)												
			L1	L2	J1	J2	H1	H2	H3	H4	H5	H6	H7	H8	H9
		Alcohols													
1	111-70-6	1-Heptanol	0.00±0.00	0.00±0.00	0.00±0.00	0.00±0.00	0.00±0.00	0.00±0.00	0.00±0.00	0.00±0.00	2.98±0.14	2.57±0.44	0.00±0.00	0.00±0.00	0.00±0.00
2	29957-43-5	Dehydrolinalool	0.00±0.00	0.00±0.00	143.76±17.73	81.85±10.93	126.78±3.63	170.67±8.25	154.17±1.88	98.77±7.77	125.63±11.88	0.00±0.00	291.3±13.65	236.73±3.84	88.54±6.05
3	143-08-8	1-Nonanol	0.00±0.00	0.00±0.00	0.00±0.00	0.00±0.00	2.81±0.72	0.00±0.00	0.00±0.00	3.62±0.96	0.00±0.00	0.00±0.00	0.00±0.00	0.00±0.00	2.28±0.13
4	14009-71-3	(Z)-linalool oxide (pyranoid)	0.00±0.00	0.00±0.00	0.00±0.00	14.27±0.38	12.53±0.61	0.00±0.00	0.00±0.00	0.00±0.00	0.00±0.00	0.00±0.00	12.44±1.02	9.14±0.46	11.5±3.03
5	1653-30-1	2-Undecanol	0.00±0.00	0.00±0.00	0.00±0.00	0.00±0.00	0.00±0.00	1.39±0	0.00±0.00	0.00±0.00	0.00±0.00	0.00±0.00	0.00±0.00	0.00±0.00	2.01±0.45
6	3809-32-3	2,5-Dimethylcyclohexanol	0.00±0.00	0.00±0.00	0.00±0.00	0.00±0.00	0.00±0.00	29.17±0.99	0.00±0.00	0.00±0.00	0.00±0.00	0.00±0.00	0.00±0.00	0.00±0.00	0.00±0.00
7	69668-82-2	3,5-Octadien-2-ol	6.75±0.18	0.00±0.00	6.08±0.88	0.00±0.00	0.00±0.00	0.00±0.00	0.00±0.00	0.00±0.00	0.00±0.00	0.00±0.00	0.00±0.00	0.00±0.00	0.00±0.00
8	29803-82-5	1-Methyl-4-(1-methylethyl)-2-cyclohexen-1-ol	0.00±0.00	0.00±0.00	0.00±0.00	0.00±0.00	5.75±0.79	0.00±0.00	0.00±0.00	0.00±0.00	0.00±0.00	0.00±0.00	0.00±0.00	0.00±0.00	0.00±0.00
9	111-76-2	2-Butoxyethanol	8.05±0.47	0.00±0.00	2.94±0.23	0.00±0.00	6.51±0.34	0.00±0.00	0.00±0.00	0.00±0.00	0.00±0.00	5.46±2	0.00±0.00	0.00±0.00	4.36±0.43
10	2425-77-6	2-Hexyl-1-decanol	0.00±0.00	0.00±0.00	0.00±0.00	19.94±11.23	0.00±0.00	0.00±0.00	0.00±0.00	18.89±11.26	5.1±0.7	0.00±0.00	0.00±0.00	0.00±0.00	0.00±0.00
11	6750-34-1	Hexahydrofarnesol	0.00±0.00	0.00±0.00	0.00±0.00	0.00±0.00	5.59±1.48	0.00±0.00	4.1±0.39	0.00±0.00	0.00±0.00	0.00±0.00	0.00±0.00	4.26±0.84	2.43±0.93
12	22627-95-8	Fenchol, exo-	0.00±0.00	0.00±0.00	0.00±0.00	5.43±4.3	4.4±1.19	6.85±1.36	0.00±0.00	3.78±1.41	4.01±1.1	5.75±2.72	7.26±3.41	10.18±0.49	9.53±0.7
13	1197-01-9	2-(4-Methylphenyl)propan-2-ol	1.71±0.17	0.00±0.00	0.00±0.00	1.44±0.09	1.78±0.14	0.00±0.00	0.00±0.00	0.00±0.00	1.38±0.08	1.4±0.14	1.62±0.17	1.97±0.13	1.78±0.16
14	78-70-6	Linalool	690.01±6.96	976.21±42.49	823.61±38.67	882.94±26.55	1098.71±61.85	758.95±19.8	728.5±8.54	950.64±1.99	761.97±20.62	709.8±34.41	709.31±10.62	662.96±9.85	788.97±17.4
15	77-53-2	Cedrol	5.33±0.92	5.88±1.51	0.00±0.00	0.00±0.00	0.00±0.00	0.00±0.00	4.47±0.74	5.1±0.05	2.77±0.03	5.03±0.29	4.73±0.56	3.78±0.26	4.2±0.59
16	39028-58-5	(E)-linalool oxide (pyranoid)	43.04±3.54	44.63±6.68	45.2±4.18	69.48±5.58	47.27±1.24	50.58±0.48	35.13±2.22	62.95±1.38	37.26±1.45	40.81±3.03	46.51±4.13	55.78±1.05	68.39±4.14
17	106-24-1	Geraniol	16.78±1.2	16.6±2.35	14.85±4.13	16.04±1.57	22.18±2.03	14.01±0.03	11.63±0.92	23.87±2.13	17.47±0.16	19.82±1.86	16.46±0.89	21.01±1.38	22.39±2.76

NO	CAS	Volatile component	Concentration (μg/kg)												
			**L1**	**L2**	**J1**	**J2**	**H1**	**H2**	**H3**	**H4**	**H5**	**H6**	**H7**	**H8**	**H9**
18	3913-2-8	2-Butyl-1-octanol	0.00±0.00	2.15±0.29	0.00±0.00	0.00±0.00	0.00±0.00	0.00±0.00	0.00±0.00	0.00±0.00	0.00±0.00	0.00±0.00	0.00±0.00	0.00±0.00	0.00±0.00
19	73741-61-4	2,4,4-Trimethyl-2-cyclohexen-1-ol	19.04±5.39	20.26±2.59	0.00±0.00	0.00±0.00	0.00±0.00	11.53±2.73	0.00±0.00	0.00±0.00	0.00±0.00	0.00±0.00	0.00±0.00	0.00±0.00	0.00±0.00
20	470-82-6	Eucalyptol	7.07±0.63	7.22±0.82	6.86±0.96	5.37±1.08	6.97±0.37	6.02±1.34	6.65±0.4	7.68±0.33	6.25±0.81	5.79±0.65	6.3±1.91	6.97±0.51	6.5±1.25
21	18409-17-1	(E)-oct-2-en-1-ol	0.00±0.00	40.04±4.46	36.96±0.95	30.41±0.42	0.00±0.00	0.00±0.00	0.00±0.00	38.86±0.19	0.00±0.00	0.00±0.00	0.00±0.00	0.00±0.00	0.00±0.00
22	507-70-0	Borneol	0.00±0.00	3.48±0.36	0.00±0.00	0.00±0.00	0.00±0.00	0.00±0.00	0.00±0.00	0.00±0.00	0.00±0.00	0.00±0.00	0.00±0.00	0.00±0.00	0.00±0.00
23	98-55-5	*α*-Terpineol	277.69±15.87	301.08±10.36	236.37±11.6	318.32±8.06	402.41±13.05	355.37±11.4	278.46±9.7	368.98±4.62	320.65±4.68	283.2±17.61	307.25±4.64	343.97±9.17	313.62±17.12
24	3391-86-4	1-Octen-3-ol	244.44±1.73	219.95±9.65	228.91±10.19	178.81±7.59	224.35±2.41	129.03±6.4	134.4±9.89	235.46±2.62	191.14±6.23	148.14±11.75	195.96±7.66	182.63±8.19	153.78±5.91
25	5989-33-3	(Z)-linalool oxide (furanoid)	68.5±6.39	80.02±5.88	65.09±1.66	50.8±2.2	63.2±3.12	47.01±5.54	39.23±0.32	59.06±1.31	39.38±2.49	42.53±4.51	53.41±0.51	62.17±1.12	61.67±9.11
26	34995-77-2	(E)-linalool oxide (furanoid)	69.6±1.46	154.76±5.91	193.09±10.35	107.82±5.07	147.98±5.21	79.64±2.13	81.26±1.19	133.55±4.08	57.32±7.68	56.78±15.9	61.27±7.93	86.67±5.64	137.72±11.89
27	26001-58-1	(2Z)-2-Octen-1-ol	69.3±3.55	0.00±0.00	0.00±0.00	0.00±0.00	38.37±0.16	13.97±2.93	16.82±3.57	0.00±0.00	33.07±0.47	31.04±0.47	40.21±0.14	39.25±2.54	27.96±3.56
28	111-87-5	1-Octanol	74.62±7.63	54.48±1.46	59.01±6.3	45.44±4.11	51.94±6.82	29.08±2.58	32.74±2.98	57.55±4.89	49.05±4.07	40.84±2.79	43.73±1.28	44.33±6.53	41.78±2.03
29	465-31-6	Camphene hydrate	13.96±0.38	14.12±0.52	12.85±1.25	13.21±0.35	15.93±1.23	12.35±0.5	10.58±0.07	15.57±0.52	12.36±0.73	10.4±0.35	13.93±0.29	13.87±0.08	12.85±0.41
30	562-74-3	Terpinen-4-ol	59.59±3.14	60.88±3.13	47.96±1.73	57.34±1.59	81.68±3.9	60.42±0.66	49.33±2.95	74.16±0.39	65.91±1.22	56.47±3.55	66.04±0.69	66.65±1.86	57.62±1.74
31	100-51-6	Benzyl alcohol	4.15±0.27	3.34±0.08	4.13±1.14	2.56±0.19	3.23±0.17	1.81±0.07	0.00±0.00	2.17±0.1	1.88±0.1	2.58±0.67	3.07±0.82	3.28±0.04	2.03±0.17
32	1632-73-1	Fenchol	16.67±2.42	14.21±0.28	12.39±1.74	0.00±0.00	0.00±0.00	0.00±0.00	0.00±0.00	0.00±0.00	0.00±0.00	0.00±0.00	0.00±0.00	0.00±0.00	0.00±0.00
		Aldehydes													
33	29548-14-9	Para-menth-1-en-9-al	7.48±0.45	0.00±0.00	0.00±0.00	0.00±0.00	0.00±0.00	0.00±0.00	0.00±0.00	0.00±0.00	0.00±0.00	3.45±1.07	0.00±0.00	0.00±0.00	0.00±0.00
34	2497-25-8	(Z)-2-decen-1-al	0.00±0.00	0.00±0.00	3.74±0.02	0.00±0.00	0.00±0.00	0.00±0.00	0.00±0.00	0.00±0.00	0.00±0.00	0.00±0.00	0.00±0.00	0.00±0.00	5.06±0.11
35	7560-64-7	4-Methylcyclohex-3-enecarbaldehyd	2.84±0.49	0.00±0.00	0.00±0.00	0.00±0.00	0.00±0.00	0.00±0.00	0.00±0.00	0.00±0.00	0.00±0.00	0.00±0.00	0.00±0.00	0.00±0.00	0.00±0.00

NO	CAS	Volatile component	Concentration (μg/kg)												
			**L1**	**L2**	**J1**	**J2**	**H1**	**H2**	**H3**	**H4**	**H5**	**H6**	**H7**	**H8**	**H9**
36	123-15-9	Methyl valeraldehyde	0.00±0.00	0.00±0.00	7.76±0.35	0.00±0.00	14.53±2.47	0.00±0.00	0.00±0.00	0.00±0.00	0.00±0.00	0.00±0.00	0.00±0.00	0.00±0.00	0.00±0.00
37	57266-86-1	2-Heptenal	30.57±0.02	0.00±0.00	0.00±0.00	0.00±0.00	0.00±0.00	0.00±0.00	0.00±0.00	10.51±1.21	0.00±0.00	11.55±2.05	15.05±1.13	17.17±2.19	14.16±0.96
38	18829-56-6	(2E)-2-Nonenal	5.03±0.39	2.7±0.63	3.41±0.17	2.14±0.02	1.98±0.48	1.45±0.02	1.59±0.11	1.97±0.12	1.81±0.68	1.79±0.3	2.01±0.5	2.24±0.17	2.9±0.41
39	15764-16-6	2,4-Dimethylbenzaldehyde	3.45±0.16	3.5±0.6	0.00±0.00	0.00±0.00	0.00±0.00	0.00±0.00	0.00±0.00	2.32±0.04	0.00±0.00	0.00±0.00	0.00±0.00	0.00±0.00	0.00±0.00
40	112-31-2	Decanal	22.7±1.13	13.9±2.31	25.16±3.31	12.32±1.69	5.25±0.72	7.09±3.26	8.72±3.63	10.09±0.64	12.74±6.66	14.43±2.13	8.62±1.45	15.47±1.65	12.72±1.75
41	2548-87-0	(2E)-2-Octenal	22.1±1.12	12.85±2.11	10.23±1.11	11.5±2.48	0.00±0.00	0.00±0.00	0.00±0.00	0.00±0.00	0.00±0.00	5.68±3.04	11.1±0.02	11.46±2.65	10.73±0.54
42	472-66-2	*β*-Homocyclocitral	11.92±1.08	12.47±1.45	10.81±1.14	7.84±1.18	8.85±1.02	5.46±0.25	7.14±0.23	9.47±0.55	8.13±0.84	5.77±0.83	7.56±1.16	6.84±0.64	8.54±0.47
43	110-62-3	Pentanal	8.95±2.13	8.86±0.92	0.00±0.00	0.00±0.00	0.00±0.00	0.00±0.00	0.00±0.00	9.7±3.05	6.73±2.47	0.00±0.00	5.95±0.98	6.31±1.44	7.7±0.23
44	3913-81-3	(2E)-2-Decenal	10.48±0.79	5.51±0.51	0.00±0.00	4.44±0.47	5.01±1.52	0.00±0.00	4.25±0.81	4.72±0.23	4.18±0.4	0.00±0.00	4.55±0.59	4.76±0.63	0.00±0.00
45	590-86-3	3-Methylbutanal	4.76±0.09	9.07±0.6	0.00±0.00	9.69±0.92	9.31±0.03	5.65±0.04	5.33±0.45	6.69±0.33	6.28±0.16	4.06±0.52	5.74±0.2	5.01±0.49	4.34±0.07
46	124-19-6	Nonanal	216.82±51.66	273.53±3.75	171.62±16.03	129±4.92	254.39±4.03	103.81±39.06	143.16±24.37	143.43±7.47	72.95±47.15	217.81±6.86	85.24±2.54	80.49±3.86	157.95±34.77
47	96-17-3	2-Methylbutanal	7.39±0.12	16.48±0.95	13.58±0.43	14.35±1.15	14.19±1.41	8.14±0.35	10.2±0.14	11.56±0.83	11.52±0.25	6.26±0.48	9.64±0.26	6.16±1.46	5.77±0.27
48	100-52-7	Benzaldehyde	76.24±7.33	117.97±4.02	119.03±4.5	113.88±2.92	98.14±8.22	47.43±0.19	52.02±3.98	80.19±0.7	79.77±2.05	52.08±3.76	74.12±2.37	47.79±6.38	41.09±1.22
49	116-26-7	Safranal	85.76±4.61	82.44±4.71	88.89±1.64	36.03±0.45	71.28±1.37	67.32±2.79	55.45±1.62	40.82±0.61	61.17±0.48	25.49±1.37	47.02±12.7	56.24±5.3	62.75±1.77
50	66-25-1	Hexanal	29.23±5.05	29.63±1.3	25.48±1.45	20.12±1.94	17.6±1.35	15.29±0.64	12.15±3.72	16.79±1.2	16.91±0.72	14.35±4.65	22.56±2.41	12.16±1.92	10.27±1.21
51	111-71-7	Heptanal	21.75±2.87	27.35±1	24.82±2.65	24.24±0.93	29.19±2.54	19.07±1.91	14.48±1.01	23.32±1.03	19.61±2.37	26.55±2.95	23.86±3.03	19.58±3.19	16.72±1.37
52	124-13-0	Octanal	50.26±0.06	41.25±2.81	0.00±0.00	30.79±1.37	38.97±1.31	0.00±0.00	0.00±0.00	33.26±1.53	0.00±0.00	25.32±0.55	30.73±0.06	26.92±4.04	27.06±2.77
53	4313-03-5	(E,E)-2,4-Heptadienal	17.72±1.18	8.79±0.24	8.94±0.95	5.6±0.5	5.69±0.19	3.09±0.33	3.72±1.01	5.67±0.64	4.37±0.07	5.01±0.25	6.38±0.15	6.03±0.11	5.89±0.3

NO	CAS	Volatile component	Concentration (μg/kg)												
			**L1**	**L2**	**J1**	**J2**	**H1**	**H2**	**H3**	**H4**	**H5**	**H6**	**H7**	**H8**	**H9**
54	122-78-1	Benzeneacetaldehyde	63.5±5.53	138.95±8.75	102.47±4.4	146.92±9.59	117.99±12.29	49.43±1.47	57.39±3.64	77.89±0.94	86.91±4.44	56.13±3.5	82.2±0.71	45.03±1.8	36.72±2.76
55	432-24-6	*α*-Cyclociral	12.19±4.52	12.67±0.19	14.89±1.65	8.56±2.4	9.29±2.25	9.96±2.62	8.94±3.93	7.25±2.24	5.89±0.28	6.72±2.47	6.75±3	10.03±0.09	10.58±3.71
56	432-25-7	*β*-Cyclocitral	112.86±11.82	120.36±9.87	116.92±2.44	98.2±3.35	105.37±9.04	114.57±3.54	92.66±1.97	105.81±1.87	96.42±1.8	69.17±1.06	98.76±1.21	91.49±8.08	106.09±3
57	6728-26-3	trans-2-Hexenal	8.19±0.04	9.99±0.09	0.00±0.00	4.59±0.49	7.57±0.46	6.87±0.16	3.35±0.28	7.72±0.24	3.05±0.28	2.68±0.3	2.9±0.1	3.6±0.44	6.48±0.4
58	18829-55-5	trans-2-Heptenal	0.00±0.00	10.85±0.07	6.21±0.27	10.13±0.6	6.59±0.51	7.08±0.55	5.15±0.23	0.00±0.00	10.4±0.78	0.00±0.00	0.00±0.00	0.00±0.00	0.00±0.00
		Alkanes													
59	2801-87-8	4-Methylpentadecane	2.9±0.01	0.00±0.00	0.00±0.00	0.00±0.00	0.00±0.00	0.00±0.00	0.00±0.00	0.00±0.00	0.00±0.00	0.00±0.00	0.00±0.00	0.00±0.00	0.00±0.00
60	13475-82-6	2,2,4,6,6-Pentamethylheptane	0.00±0.00	0.00±0.00	1.13±0.15	0.00±0.00	0.00±0.00	0.00±0.00	0.00±0.00	0.00±0.00	0.00±0.00	0.00±0.00	0.00±0.00	0.00±0.00	0.00±0.00
61	3892-00-0	2,6,10-Trimethylpentadecane	0.00±0.00	0.00±0.00	0.00±0.00	0.00±0.00	13.91±3.5	2.42±0.34	0.00±0.00	0.00±0.00	0.00±0.00	15.45±5.88	0.00±0.00	0.00±0.00	0.00±0.00
62	6165-40-8	7-Methylpentadecane	0.00±0.00	0.00±0.00	0.00±0.00	0.00±0.00	4.94±1.09	0.00±0.00	0.00±0.00	0.00±0.00	5.69±1.62	0.00±0.00	6.92±3.39	0.00±0.00	0.00±0.00
63	13151-77-4	2-Undecanylcyclohexane	0.00±0.00	0.00±0.00	0.00±0.00	0.00±0.00	5.85±1.11	8.58±2.72	3.63±0.36	0.00±0.00	0.00±0.00	0.00±0.00	0.00±0.00	0.00±0.00	0.00±0.00
64	17312-60-6	6-Ethylundecane	0.00±0.00	0.00±0.00	0.00±0.00	0.00±0.00	0.00±0.00	0.00±0.00	0.00±0.00	0.00±0.00	0.00±0.00	0.00±0.00	1.7±0.01	0.00±0.00	0.00±0.00
65	61141-72-8	Dodecane, 4,6-dimethyl-	0.00±0.00	0.00±0.00	0.00±0.00	2.66±0.87	0.00±0.00	0.00±0.00	0.00±0.00	0.00±0.00	0.00±0.00	0.00±0.00	0.00±0.00	0.00±0.00	0.00±0.00
66	295-17-0	Cyclotetradecane	0.00±0.00	0.00±0.00	9.25±1.23	0.00±0.00	0.00±0.00	0.00±0.00	0.00±0.00	0.00±0.00	0.00±0.00	0.00±0.00	0.00±0.00	0.00±0.00	0.00±0.00
67	31295-56-4	2,6,11-Trimethyldodecane	0.00±0.00	0.00±0.00	0.00±0.00	0.00±0.00	0.00±0.00	0.00±0.00	0.00±0.00	0.00±0.00	0.00±0.00	1.9±0.05	1.94±0.16	2.41±0.38	0.00±0.00
68	2882-96-4	3-Methylpentadecane	13.03±0.17	0.00±0.00	11±4.79	14.25±2.99	0.00±0.00	14.19±0.73	13.46±1.1	9.1±2.79	15.01±3.12	17.15±0.83	11.82±3.63	14.11±1.82	11.57±4.5
69	3891-98-3	2,6,10-Trimethyldodecane	2.31±0.62	0.00±0.00	0.00±0.00	0.00±0.00	7.09±0.35	0.00±0.00	2.68±0.04	0.00±0.00	0.00±0.00	0.00±0.00	0.00±0.00	4.12±0.29	0.00±0.00
70	19780-34-8	Tridecane, 3-methylene	4.2±0.16	8.1±2.46	9.76±2.77	7.08±2.36	9.4±2.5	0.00±0.00	4.8±1.35	3.68±0.02	5.9±2.93	6.2±1.6	0.00±0.00	6.78±1.37	10.33±0.46
71	2883-02-5	n-Nonylcyclohexane	10.72±0.99	8.31±2.29	9.46±1.16	11.3±1.61	9.5±1.79	7.6±0.39	7.02±0.63	9.09±1.57	10.12±0.89	10.1±1.29	5.55±2.35	8.93±0.46	9.51±1.77

NO	CAS	Volatile component	Concentration (μg/kg)												
			L1	L2	J1	J2	H1	H2	H3	H4	H5	H6	H7	H8	H9
72	111-65-9	Octane	9.29±1.12	7.4±1.51	6.28±0.17	4.47±0.07	4.15±0.48	2.6±0.08	3.12±0.1	4.9±0.1	4.64±0.14	2.55±0.08	4.65±0.11	3.2±0.61	3.68±0.1
73	2882-98-6	nonylcyclopentane	6.11±1.99	10.54±2.08	9.13±1.58	9.22±1.8	9.61±0.59	8.45±1.58	8.1±1.59	6.78±1.03	7.59±0.23	10.66±0.66	7.27±2.27	10.29±0.74	11.12±1
74	96-14-0	3-Methylpentane	3.3±0.27	4.76±0.8	3.8±0.46	0.00±0.00	3.24±0.42	3.54±0.54	3.92±0.35	3.79±0.22	0.00±0.00	0.00±0.00	0.00±0.00	0.00±0.00	0.00±0.00
75	1120-21-4	Undecane	0.00±0.00	14.47±1.98	14.66±1.59	9.08±2.67	10.26±1.31	9.2±2.29	6.27±1.23	4.36±0.2	5.06±1.7	9.89±1.93	0.00±0.00	5.85±0.85	8.76±0.75
76	6418-41-3	3-Methyltridecane	8.47±0.76	20.24±2.71	21.12±0.54	17.81±2.44	17.92±2.06	17.71±3.16	12.88±0.83	13.65±0.02	0.00±0.00	13.03±2.18	10.59±0.63	17.54±3.24	13.26±7.73
77	13287-21-3	6-Methyltridecane	0.00±0.00	6.78±0.74	0.00±0.00	6.79±0.65	0.00±0.00	4.44±0.9	2.26±0.17	0.00±0.00	0.00±0.00	2.9±0.59	0.00±0.00	3.01±0.45	0.00±0.00
78	1002-43-3	3-Methylundecane	1.66±0.09	2.95±0.25	3.35±0.11	2.28±0.14	3.07±0.3	0.00±0.00	0.00±0.00	2.12±0.22	0.00±0.00	1.36±0.14	1.53±0.16	2.02±0.54	2.34±0.31
79	111-84-2	Nonane	2.62±0.14	2.08±0.17	1.78±0.01	1.59±0.06	1.62±0.15	0.88±0.05	1.17±0.04	1.62±0.08	1.36±0.12	0.96±0.04	1.47±0.05	1.3±0.11	1.28±0.07
80	6785-23-5	Undecylcyclopentane	0.00±0.00	8.87±0.55	0.00±0.00	0.00±0.00	0.00±0.00	7.3±0.67	8.16±1.24	0.00±0.00	10.44±2.43	0.00±0.00	0.00±0.00	0.00±0.00	11.47±0.97
81	5617-41-4	Heptylcyclohexane	2.15±0.28	3.98±0.19	4.9±0.13	3.73±0.08	4.09±0.22	0.00±0.00	2.88±0.23	3±0.06	3.27±0.06	2.72±0.17	2.62±0.3	3.22±0	3.85±0.36
82	192823-15-7	Decane, 2,3,5,8-tetramethyl-	0.00±0.00	3.06±0.07	0.00±0.00	0.00±0.00	0.00±0.00	3.11±1.49	0.00±0.00	0.00±0.00	3.91±0.95	0.00±0.00	0.00±0.00	1.74±0.53	2.25±0.36
83	25117-31-1	5-Methyltridecane	0.00±0.00	2.04±0.03	0.00±0.00	0.00±0.00	0.00±0.00	0.00±0.00	0.00±0.00	0.00±0.00	0.00±0.00	0.00±0.00	0.00±0.00	0.00±0.00	1.59±0.13
		Aromatic hydrocarbons													
84	91-57-6	2-Methylnaphthalene	0.00±0.00	0.00±0.00	0.00±0.00	7.98±1.09	0.00±0.00	0.00±0.00	0.00±0.00	9.27±0.2	0.00±0.00	8.36±0.02	0.00±0.00	8.94±0.55	0.00±0.00
85	2027-17-0	2-isopropylnaphthalene	0.00±0.00	0.00±0.00	0.00±0.00	0.00±0.00	0.00±0.00	1.23±0.03	0.00±0.00	0.00±0.00	0.00±0.00	0.00±0.00	0.00±0.00	0.00±0.00	0.00±0.00
86	483-77-2	Calamenene	0.00±0.00	0.00±0.00	1.25±0.16	1.45±0.11	0.00±0.00	0.00±0.00	0.00±0.00	0.00±0.00	0.00±0.00	1.42±0.09	0.00±0.00	0.00±0.00	0.00±0.00
87	483-75-0	*α*-muurolene	1.41±0.04	0.00±0.00	0.00±0.00	0.00±0.00	0.00±0.00	0.00±0.00	0.00±0.00	0.00±0.00	0.00±0.00	0.00±0.00	0.00±0.00	0.00±0.00	0.00±0.00
88	73209-42-4	trans-Calamenene	0.00±0.00	0.00±0.00	0.00±0.00	0.00±0.00	0.00±0.00	0.00±0.00	1.31±0.02	0.00±0.00	0.00±0.00	0.00±0.00	0.00±0.00	0.00±0.00	0.00±0.00
89	95-63-6	1,2,4-Trimethylbenzene	0.00±0.00	0.00±0.00	0.00±0.00	0.00±0.00	0.00±0.00	0.00±0.00	0.00±0.00	0.00±0.00	0.00±0.00	7.03±0.17	0.00±0.00	0.00±0.00	0.00±0.00

NO	CAS	Volatile component	Concentration (μg/kg)												
			**L1**	**L2**	**J1**	**J2**	**H1**	**H2**	**H3**	**H4**	**H5**	**H6**	**H7**	**H8**	**H9**
90	538-68-1	Phenylpentane	0.00±0.00	0.00±0.00	1.99±0.03	0.00±0.00	0.00±0.00	0.00±0.00	0.00±0.00	0.00±0.00	0.00±0.00	0.00±0.00	0.00±0.00	0.00±0.00	0.00±0.00
91	941-60-6	1,1,4,6-Tetramethylindane	0.00±0.00	0.00±0.00	0.00±0.00	0.00±0.00	2.95±0.01	0.00±0.00	0.00±0.00	0.00±0.00	0.00±0.00	0.00±0.00	0.00±0.00	0.00±0.00	0.00±0.00
92	581-42-0	2,6-Dimethylnaphthalene	2.77±0.43	0.00±0.00	3.04±0.29	0.00±0.00	2.98±0.21	0.00±0.00	2.99±0.23	0.00±0.00	0.00±0.00	0.00±0.00	0.00±0.00	0.00±0.00	2.8±0.12
93	208-96-8	Acenaphthylene	0.00±0.00	0.00±0.00	0.00±0.00	0.00±0.00	0.00±0.00	2.98±0.19	0.00±0.00	0.00±0.00	1.4±0.02	2.53±0.8	0.00±0.00	0.00±0.00	0.00±0.00
94	24157-81-1	2,6-Diisopropylnaphthalene	0.00±0.00	0.00±0.00	2.02±0.13	0.00±0.00	0.00±0.00	0.00±0.00	2.6±0.67	0.00±0.00	0.00±0.00	4.54±1.43	0.00±0.00	0.00±0.00	0.00±0.00
95	575-37-1	1,7-Dimethylnaphthalene	2.52±0.22	0.00±0.00	2.98±0.3	4.24±0.49	4.25±0.56	4.16±0.1	3.47±0.65	3.48±0.3	2.89±0.23	3.68±0.57	2.61±0.21	0.00±0.00	4.34±0.09
96	1195-32-0	2-p-Tolyl-1-propene	0.00±0.00	0.00±0.00	0.00±0.00	0.00±0.00	0.00±0.00	0.00±0.00	0.00±0.00	0.00±0.00	0.00±0.00	0.00±0.00	46.18±9.22	0.00±0.00	0.00±0.00
97	61141-66-0	3,4'-Diethyl-1,1'-biphenyl	0.00±0.00	0.00±0.00	0.00±0.00	0.00±0.00	0.00±0.00	20.78±0.4	16.37±0.18	0.00±0.00	0.00±0.00	0.00±0.00	0.00±0.00	0.00±0.00	0.00±0.00
98	31983-22-9	1,2,4a,5,6,8a-Hexahydro-4,7-dimethyl-1-(1-methylethyl)napthtalene	1.81±0.08	0.00±0.00	0.00±0.00	0.00±0.00	0.00±0.00	0.00±0.00	0.00±0.00	0.00±0.00	0.00±0.00	3.66±1.2	0.00±0.00	3.47±1.45	2.07±0.09
99	575-43-9	1,6-Dimethylnaphthalene	0.00±0.00	0.00±0.00	0.00±0.00	0.00±0.00	0.00±0.00	0.00±0.00	0.00±0.00	2.72±0.27	0.00±0.00	3.74±0.4	0.00±0.00	3.19±0.49	0.00±0.00
100	92-52-4	Biphenyl	2.79±0.25	0.00±0.00	2.35±0.1	2.35±0.11	2.26±0.01	3.61±0.08	2.26±0.31	0.00±0.00	0.00±0.00	2.44±0.43	2.11±0.24	2.8±0.41	2.68±0.33
101	571-58-4	1,4-Dimethylnaphthalene	0.00±0.00	0.00±0.00	0.00±0.00	0.00±0.00	0.00±0.00	0.00±0.00	0.00±0.00	0.00±0.00	0.00±0.00	0.00±0.00	0.00±0.00	3.35±0.29	0.00±0.00
102	21391-99-1	α-Calacorene	1.15±0.04	0.00±0.00	0.00±0.00	1.15±0.09	1.38±0.18	1.36±0.01	0.00±0.00	0.00±0.00	0.00±0.00	0.00±0.00	0.00±0.00	1.36±0.07	0.00±0.00
103	2471-83-2	1-ethylidene-1H-indene	0.00±0.00	6.23±2.98	2.08±0.14	6.98±0.64	7.07±0.78	0.00±0.00	0.00±0.00	4.07±1.88	0.00±0.00	3.07±0.18	4.65±1.24	0.00±0.00	0.00±0.00
104	90-12-0	1-Methylnaphthalene	9.31±0.39	9.22±1.22	8.69±1.27	0.00±0.00	10.17±0.66	8.7±0.35	7.57±1	0.00±0.00	7.79±0.99	4.59±1.51	7.67±0.08	4.74±0.61	9.59±1.59
105	106-42-3	p-Xylene	10.19±0.41	10.31±0.94	12.09±0.07	7.98±0.42	9.65±1.06	8.57±0.58	8.11±1.3	9.79±0.54	9.19±0.73	7.29±0.33	7.82±0.38	5.59±0.25	7.28±0.95
106	100-41-4	Ethylbenzene	2.33±0.21	2.59±0.21	4.74±0.22	2.08±0.11	1.93±0.24	2.6±0.02	2.09±0.13	2.33±0.13	2.45±0.19	1.55±0.06	2.08±0.08	1.78±0.33	1.97±0.11
107	108-88-3	Toluene	12.48±1.45	14.46±0.97	23.12±1.1	11.27±0.41	12.85±1.02	9.89±0.51	10.12±0.34	13.47±0.12	12.22±0.35	7.32±0.78	9.46±0.63	9.25±0.79	7.27±0.94

NO	CAS	Volatile component	Concentration (μg/kg)												
			L1	L2	J1	J2	H1	H2	H3	H4	H5	H6	H7	H8	H9
108	99-87-6	p-Cymene	127.92±6.51	144.59±10.24	148.22±3.25	101.81±5.35	193.95±3.63	97.82±2.5	89.67±8.44	168.77±3.89	128.27±6.89	97.82±3.02	143.37±10.61	119.67±5.06	88.83±1.29
109	95-47-6	o-Xylene	5.12±0.45	4.67±0.29	6.14±0.32	3.78±0.21	3.72±0.2	4.17±0.35	4.11±0.18	4.37±0.2	3.32±0.32	2.81±0.15	3.37±0.09	3.15±0.42	3.71±0.53
110	100-42-5	Styrene	11.97±0.78	11.39±0.68	12.06±0.47	9.55±0.45	9.67±1	9.53±0.2	9.71±0.7	10.9±0.27	10.37±0.39	7.95±0.32	8.95±0.91	8.68±0.81	7.91±0.07
111	483-76-1	Δ-Cadinene	3.71±2.2	2.61±0.15	4.23±1.34	3.8±2.08	4.06±1.11	3.31±1.04	3.15±1.57	2.94±0.52	2.39±0.16	4.4±1.56	3.38±1.52	3.3±1.46	4.6±1.79
112	30364-38-6	1,2-Dihydro-1,1,6-trimethylnaphthalene	4.33±0.47	6.33±0.35	6.15±0.21	5.15±0.16	7.23±0.01	6.11±0.03	5.61±0.66	5.92±0	6.53±0.01	5.3±0.54	4.89±0.09	5.64±0.86	5.42±0.02
113	26137-53-1	1,2,3-Trimethyl-4-prop-1-enylnaphthalene	25.6±1.98	21.34±1.12	10.97±0.91	19.38±8.27	31.55±1.37	22.41±7.28	9.51±0.87	31.51±1.36	13.33±0.64	10.93±1.23	9.35±0.54	10.08±0.92	8.08±0.52
114	490-65-3	1-Methyl-7-propan-2-ylnaphthalene	0.00±0.00	2.34±0.12	2.12±0.04	1.82±0.13	1.77±0	0.00±0.00	1.97±0.04	1.92±0.01	2.24±0.04	2.02±0.31	0.00±0.00	0.00±0.00	0.00±0.00
115	575-41-7	1,3-Dimethylnaphthalene	0.00±0.00	2.86±0.12	0.00±0.00	0.00±0.00	0.00±0.00	4.52±0.85	0.00±0.00	0.00±0.00	3.82±0.3	2.58±0.1	2.5±0.05	3.79±1.28	0.00±0.00
116	91-20-3	Naphthalene	29.34±2.72	26.82±0.85	27.5±0.09	26.65±0.86	29.58±2.01	26.74±2.86	27.94±1.5	25.58±0.67	26.26±0.87	24.73±1.57	23.91±0.23	25.13±1.69	25.75±1.57
117	3075-84-1	2,2',5,5'-Tetramethylbiphenyl	16.72±1.38	13.7±0.41	25.53±0.69	16.62±3.43	15.82±4.03	15.22±2.95	11.48±2.36	18.94±0.23	17.57±4.24	16.23±1.73	12.38±3.06	13.13±1.93	12.91±2.46
118	4994-16-5	4-Phenyl-1-cyclohexene	3.5±0.25	3.98±0.11	3.83±0.16	4.58±0.3	3.58±0.23	8.27±0.52	6.27±0.89	3.78±0.05	3.7±0.09	3.4±0.32	3.38±0.06	3.98±0.61	4.04±0.15
119	526-73-8	1,2,3-Trimethylbenzene	14.78±1.29	13.12±0.32	15.68±0.99	8.8±1.24	10.87±2.06	6.97±0.44	6.59±0.37	11.75±0.82	9.01±0.87	6.52±1.25	6.62±0.8	6.87±0.6	8.22±0.66
120	72937-55-4	cis-Calamenene	0.00±0.00	1.32±0.03	0.00±0.00	0.00±0.00	3.14±0.09	1.4±0.02	0.00±0.00	1.55±0.09	1.38±0.1	0.00±0.00	1.45±0.04	3.09±1.41	0.00±0.00
121	16204-57-2	1,1,4,5-Tetramethylindane	0.00±0.00	4.89±0.05	4.54±1.29	0.00±0.00	0.00±0.00	0.00±0.00	3.9±0.56	0.00±0.00	0.00±0.00	2.32±0.13	4.39±0.11	4.25±1.08	0.00±0.00
Esters															
122	15356-74-8	Dihydroactinolide	0.00±0.00	0.00±0.00	0.00±0.00	16.92±4.93	0.00±0.00	0.00±0.00	0.00±0.00	0.00±0.00	39.02±8.25	0.00±0.00	0.00±0.00	0.00±0.00	21.33±0.68
123	93-58-3	Methyl benzoate	0.00±0.00	0.00±0.00	2.69±0.96	0.00±0.00	0.00±0.00	0.00±0.00	0.00±0.00	0.00±0.00	0.00±0.00	0.00±0.00	0.00±0.00	0.00±0.00	0.00±0.00
124	106-70-7	Methyl hexoate	5.05±1.28	0.00±0.00	5.92±1.44	0.00±0.00	0.00±0.00	3.32±0.78	0.00±0.00	0.00±0.00	0.00±0.00	0.00±0.00	0.00±0.00	0.00±0.00	0.00±0.00

NO	CAS	Volatile component	Concentration (μg/kg)												
			**L1**	**L2**	**J1**	**J2**	**H1**	**H2**	**H3**	**H4**	**H5**	**H6**	**H7**	**H8**	**H9**
125	64187-83-3	ethyl (Z)hex-3-enoate	0.00±0.00	0.00±0.00	0.00±0.00	0.00±0.00	0.00±0.00	41.56±2.4	0.00±0.00	0.00±0.00	0.00±0.00	0.00±0.00	0.00±0.00	0.00±0.00	0.00±0.00
126	97-64-3	Ethyl lactate	0.00±0.00	0.00±0.00	0.00±0.00	9.85±2.78	1.72±0.83	0.79±0.01	0.00±0.00	0.00±0.00	0.00±0.00	0.00±0.00	0.00±0.00	0.00±0.00	0.00±0.00
127	101-41-7	Methyl phenylacetate	0.00±0.00	0.00±0.00	1.7±0.08	2.57±1.18	1.99±0.67	0.00±0.00	0.00±0.00	0.00±0.00	0.00±0.00	0.00±0.00	0.00±0.00	0.00±0.00	0.00±0.00
128	31501-11-8	(3Z)-3-Hexen-1-yl hexanoate	9.97±3.18	0.00±0.00	0.00±0.00	0.00±0.00	8.77±2.01	2.89±0.12	2.65±0.37	0.00±0.00	0.00±0.00	4.76±1.06	1.72±0.01	2.8±0.13	3.68±0.18
129	84-74-2	Dibutyl phthalate	4.1±1.71	1.78±0.46	1.69±0.03	0.00±0.00	0.00±0.00	0.00±0.00	0.00±0.00	0.00±0.00	2.24±0.53	0.00±0.00	1.82±0.08	2.22±0.67	0.00±0.00
130	6846-50-0	2,2,4-Trimethyl-1,3-pentanediol diisobutyrate	102.67±6.87	90.37±5.61	83.19±12.7	90.43±8.74	95.74±13.2	69.74±3.95	59.07±10.92	75.06±4.2	94.11±3.99	85.02±4.75	76.96±19.16	89.89±1.55	76.05±17.57
131	106-32-1	Ethyl caprylate	12.17±5.99	3.83±0.72	2.54±0.07	9.47±5.03	3.54±0.69	2.77±0.07	2.88±0.67	0.00±0.00	0.00±0.00	2.66±0.24	0.00±0.00	7.19±0.38	0.00±0.00
132	111-11-5	Methyl octylate	4.94±1.5	4.37±0.73	4.47±0.79	0.00±0.00	0.00±0.00	0.00±0.00	0.00±0.00	0.00±0.00	2.99±0.54	3.73±1.59	0.00±0.00	3.08±0.59	2.68±0.84
133	112-39-0	Methyl palmitate	5.84±1.54	7.15±1.02	2.39±0.91	6.42±0.73	5.62±0.19	5.96±1.35	4.2±0.02	5.5±0.42	5.95±0.64	5.57±1.71	5.59±0.45	0.00±0.00	0.00±0.00
134	1731-84-6	Methyl nonanoate	2.33±0.09	3.65±0.35	2.51±0.05	2.71±0.1	2.13±0.12	3.56±0.18	1.76±0.09	2.43±0.02	2.03±0.04	2.07±0.15	2.55±0.42	2.36±0.2	2.64±0.42
135	123-66-0	Ethyl hexanoate	21.08±0.79	16.87±1.53	23.8±3.32	4.96±1.46	8.58±0.45	2.81±0.05	7.31±1.41	8.88±1.63	6.25±0.13	7.46±1.95	18.44±2.17	12.18±1.6	7.98±0.37
136	109-21-7	Butyl butanoate	0.00±0.00	4.32±0.38	0.00±0.00	0.00±0.00	0.00±0.00	0.00±0.00	0.00±0.00	0.00±0.00	0.00±0.00	0.00±0.00	0.00±0.00	0.00±0.00	0.00±0.00
137	590-01-2	Butyl propionate	13.65±1.1	12.39±0.96	26.81±1.12	8.22±0.45	9.16±1.16	3.57±0.19	5.12±0.33	20.82±0.9	12.27±0.67	10.83±1.08	12.48±2.26	1.44±0.05	0.00±0.00
138	119-36-8	Methyl salicylate	41.07±2.72	49.25±2.54	39.27±2.18	15.97±4.32	42.97±0.81	13.27±4.12	23.84±0.96	32.56±2.42	16.73±0.46	12.78±5.59	17.69±4.88	12.5±0.47	19.7±10.95
139	17092-92-1	Dihydroactinidiolide	56.49±2.17	33.65±1.46	35.54±0.46	36.66±2.94	30.22±4.68	29.67±4.79	28.78±7.84	28.87±7.21	29.59±0.71	28.04±0.09	38.89±4.79	36.47±9.08	25.59±3.36
140	7320-37-8	1,2-Epoxyhexadecane	0.00±0.00	0.00±0.00	0.00±0.00	0.00±0.00	15.76±1.78	0.00±0.00	0.00±0.00	0.00±0.00	0.00±0.00	0.00±0.00	0.00±0.00	0.00±0.00	0.00±0.00
141	91-16-7	1,2-Dimethoxybenzene	0.00±0.00	1.5±0.09	0.00±0.00	2.56±0.67	0.00±0.00	0.00±0.00	0.00±0.00	0.00±0.00	0.00±0.00	0.00±0.00	3.06±1.27	1.66±0.21	2.68±0.82
142	142-96-1	n-Butyl ether	0.00±0.00	4.16±0.03	5.2±0.22	0.00±0.00	0.00±0.00	1.26±0.07	0.00±0.00	0.00±0.00	0.00±0.00	0.00±0.00	2.53±0.09	0.00±0.00	0.00±0.00
		Heterocyclic compounds													

NO	CAS	Volatile component	Concentration (μg/kg)												
			**L1**	**L2**	**J1**	**J2**	**H1**	**H2**	**H3**	**H4**	**H5**	**H6**	**H7**	**H8**	**H9**
143	70424-14-5	trans-2-(2-Pentenyl)furan	0.00±0.00	0.00±0.00	45.24±2.21	0.00±0.00	0.00±0.00	0.00±0.00	0.00±0.00	0.00±0.00	0.00±0.00	0.00±0.00	0.00±0.00	0.00±0.00	0.00±0.00
144	132-64-9	Dibenzofuran	0.00±0.00	0.00±0.00	0.00±0.00	0.00±0.00	0.00±0.00	0.00±0.00	0.00±0.00	0.00±0.00	0.00±0.00	2.19±0.18	0.00±0.00	0.00±0.00	0.00±0.00
145	1487-99-6	Oxepine, 2,7-dimethyl-	2.04±0.05	0.00±0.00	0.00±0.00	0.00±0.00	1.63±0.14	0.00±0.00	0.00±0.00	1.91±0.08	0.00±0.00	1.24±0.02	0.00±0.00	0.00±0.00	0.00±0.00
146	41678-32-4	Dihydroedulan	7.4±0.96	0.00±0.00	8.84±3.23	3.46±1.34	6.94±0.38	6.04±0.89	4.25±0.04	9.26±0.28	3.95±1.61	0.00±0.00	3.03±0.4	7.65±0.24	6.48±0.99
147	1786-08-9	Neryl oxide	4.59±0.14	2.67±0.7	2.23±0.73	1.56±0.07	1.96±0.07	3.24±0.13	2.6±0.27	1.94±0.08	3.18±0.03	2.95±0.26	2.16±0.15	2.33±0.88	2.5±0.76
148	624-92-0	Methyl disulfide	2.11±0.28	1.85±0.48	1.95±0.53	2.03±0.62	1.48±0.08	1.67±0.2	1.38±0.09	1.57±0.06	1.76±0.2	0.00±0.00	1.4±0.01	0.00±0.00	1.46±0.33
149	3208-16-0	2-Ethylfuran	4.73±0.79	4.61±0.71	5.73±0.58	0.00±0.00	4.32±0.42	0.00±0.00	0.00±0.00	4.11±0.43	3.42±0.79	0.00±0.00	3.27±0.26	0.00±0.00	3±0.04
150	120-72-9	Indole	22.75±6.27	4.19±0.57	5.2±0.02	2.84±0.1	6.61±0.16	1.6±0.18	2.32±0.24	22.35±2.71	13.6±2.15	10.91±0.66	3.46±0.53	5±0.84	12.22±1.28
151	3658-80-8	Dimethyl trisulfide	25.87±1.11	21.53±2.81	17.77±0.65	21.9±3.11	10.78±0.38	11.34±3.31	8.12±2.37	12.19±0.59	14.06±1.06	6.5±0.78	10.63±2.83	5.89±2.17	9.22±2.84
152	95-16-9	Benzothiazole	37.63±1.62	35.83±3.64	26.47±6.12	29.77±7.3	38.87±1.2	32.24±10.63	41.39±1.9	33.28±1.61	33.17±3.97	34.83±3.92	28.82±3.73	49.96±3.36	41.41±1.41
153	3777-69-3	2-Pentylfuran	224.46±21.25	211.55±7.07	218.36±7.08	190.26±5.95	191.04±3.64	156.63±1.96	147.59±7.16	196.65±11.94	169.78±5.15	136.29±7.64	180.07±6.07	135.62±8.66	128.04±3.61
154	4466-24-4	2-Butylfuran	2.92±0.4	2.11±0.14	2.8±0.1	1.45±0.09	1.6±0.1	1.03±0.03	1.33±0.09	1.86±0.07	1.44±0.04	1.03±0.1	1.48±0.13	1±0.02	0.75±0.04
155	2167-14-8	1-Ethyl-1H-pyrrole-2-carbaldehyde	84.14±6.93	72.57±1.69	118.71±5.78	102.92±3.58	154.3±15.62	52.09±2.69	51.23±2.72	159.86±0.75	111.7±4.76	92.85±6.57	96.28±0.49	77.88±0.93	97.86±2.34
156	617-92-5	N-Ethylpyrrole	5.36±0.38	3.69±0.19	7.05±0.45	3.99±0.11	6.53±0.77	2.22±0.08	3.04±0.27	8.74±0.21	4.76±0.26	4.81±0.25	3.92±0.11	3.41±0.75	5.94±0.18
157	54750-69-5	(Z)-dehydroxylinalool oxide	39.73±2.93	34.19±1.71	34.73±4.86	36.73±2.03	31.26±3.41	29.37±0.94	22.93±1.74	32.23±0.59	30.86±0.77	21.24±1.16	34.59±0.49	27.26±3.49	22.17±1.25
158	36431-72-8	Theaspirane	77.9±7.27	34.08±2.24	61.59±3.02	28.77±1.96	47.6±4.42	57.96±2.85	19.19±1.66	72.72±1.37	45.05±0.7	44.72±1.46	29.89±2.59	44.28±6.1	29.79±1.36
		Ketones													
159	488-10-8	(Z)-Jasmone	9.19±0.12	0.00±0.00	0.00±0.00	0.00±0.00	5.17±1.89	0.00±0.00	0.00±0.00	10.68±0.68	7.01±1.4	7.9±0.95	0.00±0.00	8.48±0.96	11.01±1.2
160	1669-44-9	3-Octen-2-one	0.00±0.00	0.00±0.00	0.00±0.00	3.43±0.58	4.1±0.19	0.00±0.00	0.00±0.00	4.01±0.21	0.00±0.00	2.73±0.41	3.68±0.43	0.00±0.00	0.00±0.00

NO	CAS	Volatile component	Concentration (μg/kg)												
			**L1**	**L2**	**J1**	**J2**	**H1**	**H2**	**H3**	**H4**	**H5**	**H6**	**H7**	**H8**	**H9**
161	119-61-9	Benzophenone	0.00±0.00	0.00±0.00	0.00±0.00	0.00±0.00	0.00±0.00	0.00±0.00	0.00±0.00	0.00±0.00	0.00±0.00	0.00±0.00	0.00±0.00	0.00±0.00	2.81±0.71
162	502-69-2	Phytone	21.41±1.39	0.00±0.00	0.00±0.00	0.00±0.00	0.00±0.00	0.00±0.00	0.00±0.00	0.00±0.00	0.00±0.00	0.00±0.00	0.00±0.00	0.00±0.00	0.00±0.00
163	464-48-2	camphor	0.00±0.00	0.00±0.00	0.00±0.00	4.44±0.15	0.00±0.00	2.54±0.14	0.00±0.00	3.97±0.15	2.56±0.08	2.04±0.21	2.51±0.11	0.00±0.00	2.41±0.29
164	106-68-3	3-Octanone	0.00±0.00	0.00±0.00	0.00±0.00	0.00±0.00	0.00±0.00	19.07±0.6	0.00±0.00	0.00±0.00	0.00±0.00	0.00±0.00	0.00±0.00	0.00±0.00	0.00±0.00
165	1203-08-3	3-Buten-2-one, 4-(2,6,6-trimethyl-1,3-cyclohexadien-1-yl)	0.00±0.00	0.00±0.00	0.00±0.00	0.00±0.00	0.00±0.00	0.00±0.00	0.00±0.00	4.28±0.15	0.00±0.00	0.00±0.00	0.00±0.00	0.00±0.00	4.05±0.21
166	585-25-1	2,3-Octanedione	23.43±2.12	0.00±0.00	0.00±0.00	15.18±0.68	14.44±1.19	0.00±0.00	0.00±0.00	16.54±0.87	0.00±0.00	11.72±1.02	0.00±0.00	0.00±0.00	14.58±0.2
167	141-79-7	Mesityl oxide	0.00±0.00	0.00±0.00	11.58±0.53	0.00±0.00	7.58±0.52	0.00±0.00	5.3±0.87	10.63±0.81	8.78±0.2	8.4±0.74	0.00±0.00	6.73±1.4	5.55±1.45
168	689-67-8	6,10-Dimethyl-5,9-undecadien-2-one	0.00±0.00	0.00±0.00	0.00±0.00	0.00±0.00	0.00±0.00	0.00±0.00	0.00±0.00	0.00±0.00	0.00±0.00	0.00±0.00	0.00±0.00	14.75±0.68	0.00±0.00
169	38284-27-4	3,5-Octadien-2-one	21.21±2.93	9.02±3.9	22.04±1.29	9±3.54	13.01±2.26	5.54±2.38	5.94±2.38	7.49±1.5	7.77±0.04	8.32±2.32	8.87±2.21	8.52±1.48	8.64±2.99
170	1604-28-0	6-Methyl-3,5-heptadiene-2-one	12.6±2.28	7.71±2.32	9.75±3.45	5.68±2.08	4.8±0.76	6.08±1.46	0.00±0.00	4.43±0.22	3.95±0.24	6.1±2.11	7.52±3.11	7.75±1.48	7.24±0.03
171	57283-79-1	5-Ethyl-6-methyl-3-hepten-2-one	8.99±1.71	6.18±1.6	0.00±0.00	0.00±0.00	0.00±0.00	0.00±0.00	0.00±0.00	4.53±1.14	0.00±0.00	0.00±0.00	0.00±0.00	0.00±0.00	0.00±0.00
172	110-43-0	2-Heptanone	11.06±0.4	6.73±1.71	12.31±0.74	5.89±0.34	6.5±0.75	2.29±0.06	4.16±0.4	6.5±1.6	6.98±0.54	4.86±0.34	6.93±0.52	3.08±1.12	3.01±0.3
173	513-20-2	Sabina ketone	2.99±0.27	4.25±0.72	6.49±0.66	0.00±0.00	0.00±0.00	0.00±0.00	0.00±0.00	0.00±0.00	0.00±0.00	0.00±0.00	1.87±0.18	0.00±0.00	2.48±0.15
174	693-54-9	2-Decanone	19.96±0.76	17.66±2.85	13.67±0.73	18.42±1.22	18.33±1.89	15.45±0.8	14.36±1.05	16.32±0.5	14.83±0.32	15.5±2.23	17.91±1.54	18.4±0.41	19.2±1.16
175	112-12-9	2-Undecanone	3.39±0.2	3.86±0.52	3.49±0.04	4.45±0.32	4.9±0.78	4.73±0.4	3.89±0.15	3.72±0.03	3.73±0.17	4.07±0.26	4.38±0.12	6.02±0.81	4.92±0.69
176	3796-70-1	Geranylacetone	17.75±1.76	13.89±1.38	5.06±0.42	8.47±3.76	13.86±1.54	13.4±0.49	9.86±4.68	16.03±0.74	11.04±4.88	0.00±0.00	13.33±0.41	0.00±0.00	13.76±0.19
177	98-86-2	Acetophenone	51.27±9.5	36.04±3.19	30.95±0.15	34.01±0.26	17.07±1.94	33.7±10.6	45.19±6.07	23.67±8.36	30.68±8.11	23.61±5.11	34.28±3.83	25.26±6.18	36.65±5.68
178	719-22-2	2,6-Di-tert-butyl-p-benzoquinone	5.75±0.96	2.34±0.2	0.00±0.00	4.01±0.99	4±1.07	2.88±0.02	0.00±0.00	0.00±0.00	2.67±0.1	3.82±0.96	0.00±0.00	3.69±0.6	3.53±0.84

NO	CAS	Volatile component	Concentration (μg/kg)												
			**L1**	**L2**	**J1**	**J2**	**H1**	**H2**	**H3**	**H4**	**H5**	**H6**	**H7**	**H8**	**H9**
179	14901-07-6	β-Ionone	91.14±2.76	95.29±3.9	92.54±6.82	100.43±3.06	90.85±6.54	102.88±5.09	74.57±1.85	80.93±0.6	89.12±5.53	71.98±7.32	78.53±4.61	82.59±5.6	86.87±3.34
180	127-41-3	α-Ionone	22.94±1.12	31.51±1.97	27.11±2.28	27.65±4.02	29.18±3.52	19.78±0.7	23±1.39	22.98±2.57	29.44±2.73	21.58±1.78	22.77±3.09	21.43±1.63	23.07±1.82
181	18402-82-9	(E)-Oct-3-en-2-one	0.00±0.00	8.46±0.46	0.00±0.00	0.00±0.00	0.00±0.00	0.00±0.00	0.00±0.00	0.00±0.00	0.00±0.00	0.00±0.00	0.00±0.00	0.00±0.00	0.00±0.00
182	110-93-0	6-Methylhept-5-en-2-one	59.06±4.95	38.26±0.68	50.68±3.57	29.83±0.92	33.75±0.7	30.06±1.29	31.14±1.66	39.15±0.93	29.74±1.71	24.01±1.63	32.75±1.42	30.2±3.03	32.07±1.81
183	30086-02-3	(E,E)-3,5-octadien-2-one	39.67±4.49	31.38±4.51	26.16±0.22	15.17±11.74	8.61±1.4	5.71±0.96	6.86±1.28	8.17±1.9	8.35±2.52	16.3±10.45	13.88±6.87	18.97±5.5	18.79±10.68
184	928-68-7	6-Methyl-2-heptanone	3.65±0.33	3.04±0.07	0.00±0.00	3.25±0.03	2.35±0.08	0.00±0.00	2.78±0.17	2.97±0.11	2.42±0.47	2.14±0.01	2.65±0.17	1.82±0.23	0.00±0.00
185	2408-37-9	2,2,6-Trimethylcyclohexan-1-one	156.17±12.21	144.32±6.31	149.62±5.34	119.73±5.09	125.27±6.76	152.37±6	151.54±12.86	142.17±1.21	124.33±4.84	90.46±2.87	140.33±2.81	104.58±8.32	123.7±4.47
186	23726-93-4	*β*-Damascenone	3.02±0.19	2.87±0.04	2.83±0.1	2.5±0.12	3.16±0.17	2.47±0.08	2.31±0.04	2.38±0.01	2.16±0.01	1.71±0.04	2.55±0.01	2.51±0.07	2.39±0.12
187	1193-18-6	3-Methyl-2-cyclohexen-1-one	0.00±0.00	39.87±0.24	0.00±0.00	26.93±1.57	0.00±0.00	0.00±0.00	30.08±2.16	29.68±3.7	25.7±1.48	20.7±0.34	30.89±1.88	27.3±1.11	28.73±1.63
		Olefins													
188	41446-67-7	(Z)-Tetradec-3-ene	0.00±0.00	0.00±0.00	0.00±0.00	0.00±0.00	0.00±0.00	2.6±0.95	0.00±0.00	0.00±0.00	0.00±0.00	0.00±0.00	0.00±0.00	0.00±0.00	0.00±0.00
189	65378-76-9	1,2,4,4-Tetramethylcyclopentene	1.59±0.03	0.00±0.00	1.63±0.12	0.00±0.00	0.00±0.00	0.00±0.00	0.00±0.00	0.00±0.00	0.00±0.00	0.00±0.00	0.00±0.00	0.00±0.00	0.00±0.00
190	1002-33-1	1,3-Octadiene	2.03±0.65	0.00±0.00	0.00±0.00	0.00±0.00	0.00±0.00	0.00±0.00	0.00±0.00	0.00±0.00	0.00±0.00	0.00±0.00	0.00±0.00	0.00±0.00	0.00±0.00
191	23051-84-5	(E)-Tridec-5-ene	3.51±0.94	0.00±0.00	0.00±0.00	0.00±0.00	0.00±0.00	0.00±0.00	0.00±0.00	0.00±0.00	0.00±0.00	0.00±0.00	0.00±0.00	0.00±0.00	0.00±0.00
192	14919-01-8	trans-3-Octene	1.5±0.06	0.00±0.00	1±0.01	0.00±0.00	0.00±0.00	0.00±0.00	0.00±0.00	0.00±0.00	0.00±0.00	0.00±0.00	0.00±0.00	0.00±0.00	0.00±0.00
193	99-84-3	*β*-Terpinene	0.00±0.00	0.00±0.00	2.34±0.08	0.00±0.00	0.00±0.00	0.00±0.00	0.00±0.00	0.00±0.00	0.00±0.00	0.00±0.00	0.00±0.00	0.00±0.00	0.00±0.00
194	500-00-5	4-Methyl-1-propan-2-ylcyclohexene	0.00±0.00	0.00±0.00	0.00±0.00	0.00±0.00	1.85±0.52	0.00±0.00	0.00±0.00	0.00±0.00	0.00±0.00	0.00±0.00	0.00±0.00	0.00±0.00	0.00±0.00
195	469-61-4	(-)-α-Cedrene	5.23±0.26	0.00±0.00	9.34±0.66	7.25±0.13	7.8±0.39	0.00±0.00	6.54±0.04	8.26±0.03	7.91±0.03	6.07±0.5	0.00±0.00	0.00±0.00	0.00±0.00
196	41446-58-6	(2E)-2-tridecene	0.00±0.00	0.00±0.00	0.00±0.00	4.03±0.72	0.00±0.00	0.00±0.00	0.00±0.00	0.00±0.00	0.00±0.00	2.73±0.24	0.00±0.00	0.00±0.00	6.83±2.1

NO	CAS	Volatile component	Concentration (μg/kg)												
			L1	L2	J1	J2	H1	H2	H3	H4	H5	H6	H7	H8	H9
197	7206-15-7	(E)-4-Dodecene	0.00±0.00	0.00±0.00	0.00±0.00	0.00±0.00	0.00±0.00	0.00±0.00	0.00±0.00	0.00±0.00	0.00±0.00	0.00±0.00	0.00±0.00	4.72±1.01	0.00±0.00
198	5989-27-5	Limonene	819.02±13.39	925.18±57.82	861±29.15	816.01±33.98	1149.42±35.21	854.96±22.82	789.65±42.78	1068.18±17.16	946.83±25.26	813.27±12.98	859.18±10.08	857.08±39.74	815.56±13.47
199	1120-36-1	1-Tetradecene	4.7±0.43	8.54±2.82	7.67±2.36	9.3±2.71	7.83±1.77	4.78±0.1	6.42±1.92	5.55±1.51	5.93±1.32	6.85±0.75	7.83±0.16	8.14±0.48	7.95±1.33
200	11028-42-5	Cedrene	0.00±0.00	3.05±0.98	3.39±0.11	3.2±0.18	0.00±0.00	0.00±0.00	0.00±0.00	3.1±0.09	2.99±0.01	0.00±0.00	5.34±0.16	5.16±0.03	5.7±0.11
201	40087-62-5	(3Z,5Z)-1,3,5-Octatriene	0.00±0.00	2±0.6	2.91±0.03	2.12±0.57	1.47±0.26	0.00±0.00	0.00±0.00	2.3±0.54	2.58±0.14	1.11±0.07	1.99±0.53	2.17±0.77	1.83±0.46
202	41446-57-5	(E)-Tridec-3-ene	0.00±0.00	9.15±2.37	0.00±0.00	0.00±0.00	7.51±1.2	5.48±0.08	0.00±0.00	0.00±0.00	0.00±0.00	0.00±0.00	0.00±0.00	0.00±0.00	0.00±0.00
203	504-96-1	Neophytadiene	10.25±2.33	7.7±1.85	8.89±4.03	12.78±2.87	6.3±0.1	25.58±0.95	2.88±0.62	9.86±2.7	10.18±3.06	15.6±12.75	5.46±1.49	5.87±0.03	5.38±0.74
204	14912-44-8	Ylangene	0.00±0.00	2.04±0.45	0.00±0.00	0.00±0.00	0.00±0.00	0.00±0.00	0.00±0.00	0.00±0.00	0.00±0.00	0.00±0.00	0.00±0.00	1.63±0.14	0.00±0.00
205	19945-61-0	(E)-4,8-dimethyl-1,3,7-nonatriene	3.49±0.54	3.67±0.55	4.15±0.06	1.91±0.17	2.95±0.09	2.28±0.19	2.82±0.37	3.32±0.16	3.23±0.25	3.99±0.99	2.27±0.09	2.56±0.19	3.66±0.32
206	13360-61-7	1-Pentadecene	0.00±0.00	12.41±1.55	0.00±0.00	14.86±3.24	0.00±0.00	0.00±0.00	10.17±0.8	0.00±0.00	10.5±0.49	10.56±0.16	9.89±2.98	11.04±0.87	16.31±4.87
207	13466-78-9	3-Carene	5.18±1.13	5.22±0.6	5.2±0.22	3.87±0.51	4.46±0.54	5.07±0.3	3.97±0.01	4.83±0.22	4.21±0.13	3.71±0.02	3.87±0.08	3.95±0.71	3.88±0.25
208	7216-56-0	Allo-ocimene	3.43±0.47	3.24±0.33	3.12±0.2	2.86±0.12	4.15±0.21	2.44±0.08	0.00±0.00	4.07±0.05	3.47±0.1	3.24±0.13	2.63±1.3	3.32±0.05	2.95±0.17
209	87-44-5	Caryophyllene	3.05±0.09	5.15±0.36	6.03±0.04	6.43±1.58	6.59±1.58	9.06±0.41	9.34±0.44	5.8±0.02	5.2±0.15	7.52±1.04	6.6±1.19	6.95±1.7	8.98±0.08
210	79-92-5	Camphene	24.39±2.42	26.51±1.73	21.92±0.51	19.3±1.31	33.47±1.48	17.89±0.54	17.4±1.47	30.77±1.17	24.61±0.88	21.31±0.58	24.27±0.99	24.19±4.26	17.82±0.59
211	123-35-3	*β*-Myrcene	70.32±3.53	84.74±5.33	78.34±3.1	71.29±3.43	118.77±1.49	60.25±3.84	64.41±7.52	105.94±3.44	92.15±1.97	74.51±1.34	74.65±3.25	71.9±2.39	67.01±2.69
212	99-85-4	*γ*-Terpinene	95.84±7.43	102.34±6	97.81±2.16	88.29±3.94	148.24±2.8	76.98±9.3	79.7±6.15	127.92±12.88	115.4±1.87	103.3±1.76	106.38±2.04	111.48±5.26	87.99±1.84
213	586-62-9	Terpinolene	158.47±24.05	163.23±7.7	138.76±3.4	128.15±7.66	225.47±7.48	121.86±2.25	131.35±3.63	205.85±4.95	185.88±13.78	173.01±14.44	175.04±10.63	204.09±28.02	138.48±12.63
214	99-86-5	α-Terpinene	78.86±7.51	85.59±4.98	75.24±2.42	67.16±3.61	120.75±4.1	61.41±1.47	59.29±5.05	112.52±3.8	90.3±3.49	79.12±0.89	85.03±1.23	76.72±2.54	63.58±1.91

NO	CAS	Volatile component	Concentration (μg/kg)												
			**L1**	**L2**	**J1**	**J2**	**H1**	**H2**	**H3**	**H4**	**H5**	**H6**	**H7**	**H8**	**H9**
215	4249-12-1	1,3-Cyclopentadiene, 1,2,5,5-tetramethyl-	24.46±0.76	24.42±1.22	27.63±0.57	19.32±1.18	20.44±1.56	0.00±0.00	0.00±0.00	22.95±0.83	21.33±1	13.75±0.29	19.97±0.83	16.35±3.04	17.72±0.49
216	99-83-2	α-Phellandrene	29.69±4.1	32.28±1.54	29.36±1.44	26.02±0.84	41.23±4.85	23.93±0.54	22.06±1.18	40.74±1.44	33.74±1.46	29.92±0.86	31.61±0.24	30.32±1.36	23.72±0.44
217	2609-23-6	2,6-Dimethylocta-2,6-diene	17.73±1.27	19.46±0.92	18.59±0.59	17.21±0.7	25.14±3.07	11.19±0.51	14.04±1.04	24.94±0.76	22.42±0.77	18.59±0.3	15.85±0.37	15.36±0.46	14.83±0.25
218	546-28-1	*β*-Cedrene	0.00±0.00	3.41±0.14	0.00±0.00	0.00±0.00	3.38±0.3	0.00±0.00	0.00±0.00	0.00±0.00	0.00±0.00	0.00±0.00	0.00±0.00	0.00±0.00	0.00±0.00
219	3779-61-1	trans-*β*-Ocimene	66.84±7.94	86.62±3.41	80.68±4.1	77.62±0.53	117.17±10.61	57.16±1.73	66.73±4.01	116.18±1.93	98.44±2.38	86.18±0.19	81.75±1.17	80.44±2.01	73.47±2.01
220	80-56-8	α-Pinene	24.43±2.11	29.43±1.11	25.79±0.96	23.4±1.35	28.48±3.22	22.82±0.85	22.34±2.1	29.81±1.36	26.81±0.86	23.12±1	22.93±1.08	24.58±4.97	21.92±0.97
221	460-01-5	Cosmene	6.4±0.32	4.42±0.16	6.65±1.57	4.28±0.89	4.69±0.21	3.72±0.25	3.42±0.08	4.57±0.52	4.35±0.42	3.68±0.19	5.36±1.15	5.32±0.71	4.37±0.3
222	26456-76-8	3,5,5-Trimethyl-2-hexene	56.44±0.93	37.76±0.91	39.99±1.36	29.86±1.27	39.55±2.98	17.26±1.03	20.09±1.72	43.03±1.98	33.69±0.48	27.26±2.13	34.17±1.4	30.76±2.75	23.42±1.99
223	629-73-2	Cetene	0.00±0.00	5.66±0.18	0.00±0.00	0.00±0.00	0.00±0.00	4.04±0.23	0.00±0.00	0.00±0.00	4.03±0.16	0.00±0.00	0.00±0.00	2.69±1.03	4.53±1.62
224	1137-12-8	longicyclene	4.41±1.7	4.64±0.11	5.34±0.8	4.56±0.9	5.56±1.28	4.25±0.1	4.18±0.07	3.39±0.09	3.6±0.05	5.49±0.22	2.86±0.22	4.71±0.7	4.85±1.26
225	13877-91-3	β-Ocimene	48.78±1.43	52.22±1.17	49.32±1.73	44.24±3.15	74.79±3	35.89±3.66	41.6±0.88	72.26±1.68	59.85±3.4	49.02±2.78	48.29±2.88	52.33±4.35	43.22±2.56
226	475-20-7	Longifolene	27.63±2.51	49±0.9	51.51±1.13	44.91±1.46	46.36±1.97	47.36±2.15	35.57±2.3	39.85±1.25	38.79±2.08	35.15±1.84	32.13±1.87	36.65±4.84	38.33±0.94
227	2792-39-4	(6E)-2,6-dimethylocta-2,6-diene	15.51±1.2	17.8±0.05	17.19±0.35	16.1±0.41	23.16±2.24	10.02±0.25	12.87±0.93	22.85±0.58	20.8±0.86	17.01±0.61	14.32±0.3	16.17±3.25	14.05±0.15
228	128-37-0	Butylated Hydroxytoluene	1.29±0.02	0.00±0.00	0.00±0.00	0.00±0.00	0.00±0.00	0.00±0.00	0.00±0.00	0.00±0.00	0.00±0.00	0.00±0.00	0.00±0.00	0.00±0.00	0.00±0.00
229	108-95-2	Phenol	0.00±0.00	0.00±0.00	0.00±0.00	0.00±0.00	0.00±0.00	0.00±0.00	0.00±0.00	0.00±0.00	2.51±0.27	0.00±0.00	0.00±0.00	0.00±0.00	0.00±0.00
230	96-76-4	2,4-Di-t-butylphenol	7.35±0.86	5.25±0.22	5.75±0.32	5.13±1.62	5±0.18	4.79±0.26	4.51±0.08	4.53±0.13	0.00±0.00	6.17±1.52	5.8±1.24	6.22±0.97	9.04±1.89

Table S2 Identification of non-volatile compounds in Sun-Dried Green Tea

Formula	m/z	RT (min)	Adducts	Name
		Alkaloids		
C9 H12 N4 O3	225.09759	8.085	[M+H]+1	Theacrine
C7 H8 N4 O2	181.07153	5.577	[M+H]+1	Theobromine
C6 H6 N4 O2	167.05597	4.362	[M+H]+1	7-Methylxanthine
C8 H10 N4 O2	195.08707	8.861	[M+H]+1	Caffeine
C10 H9 N	144.08054	6.191	[M+H]+1	6-Methylquinoline
		Amino acids and their derivatives		
C6 H13 N O2	132.10167	1.88	[M+H]+1	Leucine
C5 H10 N2 O3	145.06203	0.802	[M-H]-1	L-glutamine
C7 H11 N O5	188.05656	0.944	[M-H]-1	N-Acetyl-DL-glutamic acid
C5 H9 N O2	116.07038	0.914	[M+H]+1	Proline
C5 H11 N O2	118.08591	0.884	[M+H]+1	L-Valine
C4 H9 N O2	104.07044	0.849	[M+H]+1	Gamma-Aminobutyric acid
C7 H14 N2 O3	173.09313	1.276	[M-H]-1	L-Theanine
C5 H7 N O3	128.03545	1.447	[M-H]-1	Pyroglutamic Acid
C5 H9 N O4	146.046	0.827	[M-H]-1	L-Glutamic acid
C19 H30 N4 O11	489.18257	0.918	[M-H]-1	Glu-glu-val-asp
C6 H9 N3 O2	156.0765	7.152	[M+H]+1	L-Histidine
C4 H7 N O4	134.04416	0.818	[M+H]+1	L-(+)-Aspartic acid
C4 H7 N O4	132.03038	0.81	[M-H]-1	L-Aspartic acid
C6 H14 N4 O2	175.11867	0.804	[M+H]+1	Arginine
C11 H20 N2 O3	229.15434	1.556	[M+H]+1	Prolylleucine
C4 H9 N O3	120.06529	0.823	[M+H]+1	L-Threonine
C20 H32 N6 O12 S2	611.14561	0.944	[M-H]-1	L-Glutathione oxidized
C6 H13 N O2	132.10169	1.738	[M+H]+1	Isoleucine
C9 H11 N O2	164.07173	4.013	[M-H]-1	L-Phenylalanine
C14 H14 N2 O5	291.09708	14.061	[M+H]+1	Indole-3-acetyl-L-aspartic acid
C13 H14 N2 O3	245.09324	14.062	[M-H]-1	N-Acetyltryptophan
C11 H12 N2 O2	203.08268	6.187	[M-H]-1	Tryptophan
C9 H11 N O3	180.06674	1.957	[M-H]-1	L-Tyrosine
		Catechins and their derivatives		
C23 H20 O11	471.09344	16.764	[M-H]-1	4''-Methylepigallocatechin Gallate
C30 H26 O12	577.13533	9.64	[M-H]-1	Procyanidin B1
C23 H20 O11	471.09344	15.332	[M-H]-1	Epigallocatechin 3-O- (3-O-methylgallate)
C15 H14 O6	289.07168	8.301	[M-H]-1	Catechin
C22 H18 O11	459.09154	13.045	[M+H]+1	Gallocatechin gallate
C22 H18 O11	457.0782	14.474	[M-H]-1	Epigallocatechin 7-O-gallate
C15 H14 O6	289.07168	11.149	[M-H]-1	Epicatechin
C22 H18 O10	443.09494	16.321	[M+H]+1	Catechin gallate
C22 H18 O11	457.07747	11.886	[M-H]-1	Epigallocatechin gallate
C22 H18 O10	441.08248	16.323	[M-H]-1	Epicatechin gallate
C30 H26 O12	579.14894	8.302	[M+H]+1	Procyanidin B4
C37 H30 O17	745.14155	10.549	[M-H]-1	Epigallocatechin-(4 β ->8)-epicatechin-3-O-gallate ester
C43 H32 O20	867.14223	23.459	[M-H]-1	Theaflavin-3,3'-digallate
C30 H26 O14	609.12535	4.344	[M-H]-1	Prodelphinidin B4
C29 H22 O15	609.09029	18.222	[M-H]-1	(-)-Epigallocatechin 3,5-digallate

Formula	m/z	RT (min)	Adducts	Name
C29 H22 O14	593.09459	21.202	[M-H]-1	Epicatechin 3,5-digallate
C30 H26 O13	593.13056	12.165	[M-H]-1	(+)-catechin-(4 α->8)-(-)-epigallocatechin
C30 H26 O14	609.12526	8.327	[M-H]-1	Prodelphinidin B3
C45 H38 O18	865.19876	10.673	[M-H]-1	Procyanidin C1
C30 H26 O12	577.13559	17.978	[M-H]-1	Procyanidin B3
C24 H20 O9	451.10385	21.933	[M-H]-1	Epigallocatechin 3-O-p-coumarate
C30 H26 O12	577.13546	10.424	[M-H]-1	(+)-Procyanidin B2
C36 H28 O16	715.13102	23.2	[M-H]-1	theaflavin 3-gallate
C44 H34 O20	883.17017	17.504	[M+H]+1	Procyanidin B2 3,3'-di-O-gallate
C37 H30 O18	761.13677	8.802	[M-H]-1	Prodelphinidin B 2, 3â€²-O-gallate
C30 H26 O13	593.13048	5.514	[M-H]-1	(+)-gallocatechin-(4 α->8)-(-)-epicatechin
C25 H22 O10	481.11457	21.597	[M-H]-1	Epigallocatechin-3-O-ferulate
C30 H26 O14	609.12576	10.181	[M-H]-1	Theasinensin C
			Flavonoids	
C21 H20 O13	479.08315	15.581	[M-H]-1	Myricetin 3-O-glucoside
C26 H28 O14	563.14199	15.012	[M-H]-1	Isoschaftoside
C42 H46 O21	442.11946	22.873	[M-2H]-2	Kaempferol 3- (4"- (E) -p-coumarylrobinobioside) -7-rhamnoside
C20 H18 O11	433.07739	19.843	[M-H]-1	Quercetin 3-O- α-L-arabinopyranside
C27 H30 O16	609.14609	18.07	[M-H]-1	Rutin
C15 H12 O5	273.07535	8.302	[M+H]+1	Naringenin
C15 H12 O5	273.07541	17.614	[M+H]+1	(-) -Glycinol
C27 H30 O15	593.15293	16.809	[M-H]-1	Nictoflorin
C15 H12 O7	303.05137	14.118	[M-H]-1	(-)-taxifolin
C36 H36 O17	741.20116	22.877	[M+H]+1	Kaempferol-3-O-(6'"-trans-P-coumaroyl-2"-glucosyl)rhamnoside
C44 H36 O22	915.16211	11.874	[M-H]-1	Procyanidin C2 3,3"-di-O-gallate
C15 H12 O6	289.07012	7.955	[M+H]+1	(+)-Aromadendrin
C30 H26 O13	593.13043	7.709	[M-H]-1	Tiliroside
C15 H12 O5	273.07523	16.312	[M+H]+1	naringenin chalcone
C33 H40 O19	741.22032	20.228	[M+H]+1	Kaempferol 3-rhamnosyl- (1->3) -rhamnosyl- (1->6) -glucoside
C27 H32 O14	579.17427	14.067	[M-H]-1	Narirutin
C39 H32 O15	739.16716	25.103	[M-H]-1	Kaempferol 3- (3",6"-di-p-coumarylglucoside)
C15 H10 O7	303.0494	17.668	[M+H]+1	Quercetin
C15 H10 O8	319.04427	15.582	[M+H]+1	Myricetin
C27 H26 O15	589.12051	15.799	[M-H]-1	Quercetin 3- (2",3",4"-triacetylgalactoside)
C26 H28 O14	565.15417	15.005	[M+H]+1	Schaftoside
C15 H10 O6	287.0545	21.94	[M+H]+1	Luteolin
C28 H24 O15	599.10541	18.816	[M-H]-1	Isoorientin 2"-O-gallate
C27 H30 O15	595.16482	16.548	[M+H]+1	Keracyanin
C15 H18 O9	341.08774	7.279	[M-H]-1	Flavoroseoside
C15 H12 O6	289.07008	15.333	[M+H]+1	Eriodictyol
C37 H30 O18	761.13585	18.426	[M-H]-1	Theasinensin B
C28 H24 O16	615.09973	17.327	[M-H]-1	Quercetin 3- (2"-galloylgalactoside)
C31 H28 O13	607.14652	15.684	[M-H]-1	Vitexin 2"-O- (E) -ferulate
C27 H22 O14	569.09452	13.946	[M-H]-1	Kaempferol 3- (2"-galloyl- α-L-arabinopyranoside)
C30 H26 O13	593.13086	6.481	[M-H]-1	Kaempferol 3- (6"-p-coumarylgalactoside)
C28 H24 O15	599.10443	20.354	[M-H]-1	Kaempferol 7- (6"-galloylglucoside)

Formula	m/z	RT (min)	Adducts	Name
C21 H20 O10	433.11235	16.734	[M+H]+1	Vitexin
C27 H30 O15	595.16287	20.405	[M+H]+1	Kaempferol 3-O-robinobioside
C15 H10 O6	287.05445	19.894	[M+H]+1	Fisetin
C15 H10 O7	303.04935	18.044	[M+H]+1	Herbacetin
C30 H28 O13	595.14634	8.286	[M-H]-1	Okanin 4'- (6"-p-coumarylglucoside)
C21 H20 O11	449.10725	19.235	[M+H]+1	Isoorientin
C33 H40 O19	739.20997	20.223	[M-H]-1	Kaempferol 3-rhamninoside
C15 H10 O7	303.04941	20.732	[M+H]+1	2-(2,4-dihydroxyphenyl)-3,5,7-trihydroxy-4H-chromen-4-one
C20 H18 O12	449.07286	10.683	[M-H]-1	Quercetin 4'-galactoside
C15 H12 O5	273.07546	21.275	[M+H]+1	Butein
C15 H10 O6	287.05445	20.675	[M+H]+1	Kaempferol
C21 H20 O12	465.102	17.668	[M+H]+1	Hyperoside
C27 H30 O15	595.16494	16.801	[M+H]+1	2"-O-(β -D-glucosyl)isovitexin
C27 H30 O17	627.15275	15.458	[M+H]+1	Quercetin 3-O-sophoroside
C15 H10 O7	301.0355	21.714	[M-H]-1	Tricetin
C21 H20 O11	449.10711	20.405	[M+H]+1	Astragalin
C42 H46 O22	901.2412	22.928	[M-H]-1	Isovitexin 2"-O- (6"'- (E) -p-coumaroyl) glucoside 4'-O-glucoside
C20 H18 O10	417.08314	21.158	[M-H]-1	Kaempferol 3- α -D-arabinopyranoside
C29 H26 O16	629.11587	21.569	[M-H]-1	Isorhamnetin 3- (6"-galloylglucoside)
C27 H30 O16	611.1578	17.674	[M+H]+1	Quercetin 3-O-rhamnoside-7-O-glucoside
C21 H18 O13	477.06797	17.505	[M-H]-1	Miquelianin
C25 H24 O11	499.12505	21.652	[M-H]-1	Apigenin 7- (6"-crotonylglucoside)
C33 H40 O21	771.19925	15.218	[M-H]-1	Quercetin 3-sophoroside-7-rhamnoside
C23 H22 O13	505.09907	15.049	[M-H]-1	6-Methoxyluteolin 7-glucuronide methyl ester
C33 H40 O19	739.20946	19.78	[M-H]-1	Kaempferol-3-Galactoside-6"-Rhamnoside-3"'-Rhamnoside
C21 H20 O11	449.10722	18.072	[M+H]+1	Quercitrin
C22 H16 O15 S	553.02933	12.031	[M+H]+1	Myricatin
C30 H26 O13	593.13064	23.204	[M-H]-1	Kaempferol-3-Glucoside-2"-p-coumaroyl
C21 H20 O11	447.09314	20.676	[M-H]-1	Trifolin
C15 H10 O7	303.04995	18.436	[M+H]+1	Morin
C27 H30 O17	625.14157	15.459	[M-H]-1	Myricetin-3-O-rutinoside
C15 H12 O6	289.0703	13.752	[M+H]+1	(-)-Fustin
C20 H18 O11	433.07812	20.247	[M-H]-1	Reynoutrin
			Lipids	
C19 H38 O4	331.284	30.819	[M+H]+1	1-palmitoyl-sn-glycerol
C18 H28 O3	291.19695	28.809	[M-H]-1	12-oxo Phytodienoic Acid
C18 H30 O2	279.23143	32.046	[M+H]+1	α -Linolenic acid
C21 H42 O4	359.31533	31.172	[M+H]+1	1-Stearoylglycerol
C18 H35 N O	282.27874	33.795	[M+H]+1	Oleamide
C18 H32 O2	281.24731	33.052	[M+H]+1	Linoleic Acid
C18 H34 O5	329.23369	24.551	[M-H]-1	(15Z)-9,12,13-Trihydroxy-15-octadecenoic acid
C18 H30 O3	295.22642	29.862	[M+H]+1	9-Oxo-ODE
C18 H30 O3	293.2125	28.441	[M-H]-1	13(S)-HOTrE
C10 H20 O4	203.12892	21.358	[M-H]-1	(3R,5R)-3,5-dihydroxydecanoic acid
			nucleotides and their derivatives	
C5 H5 N5	136.06149	2.963	[M+H]+1	Adenine

Formula	m/z	RT (min)	Adducts	Name
C10 H14 N5 O8 P	362.05081	1.844	[M-H]-1	Guanosine monophosphate (GMP)
C9 H12 N2 O6	243.06237	1.741	[M-H]-1	Uridine
C9 H13 N2 O9 P	323.02863	0.922	[M-H]-1	Uridine monophosphate (UMP)
C10 H15 N5 O10 P2	426.02195	0.922	[M-H]-1	Adenosine diphosphate
C11 H15 N5 O4 S	314.09128	3.054	[M+H]+1	5'-Deoxy-5'-[(R)-methylsulfinyl]adenosine
C5 H5 N5 O	152.05677	3.521	[M+H]+1	Guanine
C10 H13 N5 O5	282.08456	3.541	[M-H]-1	Guanosine
C10 H12 N5 O6 P	330.05929	1.788	[M+H]+1	Adenosine 3'5'-cyclic monophosphate
C10 H13 N5 O4	268.10347	2.965	[M+H]+1	Adenosine
C10 H12 N4 O6	283.06854	4.617	[M-H]-1	Xanthosine
C10 H14 N5 O7 P	348.0688	1.333	[M+H]+1	Adenosine 5'-monophosphate
C10 H14 N5 O7 P	348.06987	1.613	[M+H]+1	3'-Adenosine monophosphate (3'-AMP)
C10 H12 N5 O7 P	344.04011	2.26	[M-H]-1	Cyclic 3',5'-guanosine monophosphate
			Organic acids	
C9 H16 O4	187.09768	18.485	[M-H]-1	Azelaic acid
C4 H6 O4	117.0195	1.611	[M-H]-1	Succinic acid
C3 H4 O4	103.00387	0.959	[M-H]-1	Malonic acid
C4 H8 O5	135.03003	0.858	[M-H]-1	L-Threonic acid
C7 H10 O5	173.04557	11.122	[M-H]-1	(-)-Shikimic acid
C7 H6 O2	123.04387	17.614	[M+H]+1	Benzoic acid
C6 H8 O7	191.01975	1.399	[M-H]-1	Citric acid
C9 H8 O4	179.03498	9.025	[M-H]-1	Phenylmalonic acid
C6 H6 O6	173.00918	0.932	[M-H]-1	trans-Aconitic acid
C4 H4 O4	115.00382	1.059	[M-H]-1	Fumaric acid
C5 H10 O6	165.04051	0.835	[M-H]-1	Ribonic acid
C5 H4 O3	111.00891	0.936	[M-H]-1	3-Furoic acid
C7 H6 O5	171.02841	4.503	[M+H]+1	（Z,Z)-4-oxo-2,5-hetpadienedioic acid
C6 H11 N O2	147.11264	0.729	[M+NH4]+1	Pipecolic acid
C7 H12 O6	191.05606	0.905	[M-H]-1	D-(-)-Quinic acid
C6 H12 O7	195.05108	0.824	[M-H]-1	Gluconic acid
C6 H10 O8	209.03036	0.832	[M-H]-1	D-Saccharic acid
C4 H6 O5	133.01436	0.943	[M-H]-1	D-(+)-Malic acid
C6 H8 O7	191.01974	1.285	[M-H]-1	Isocitric acid
C11 H9 N O2	188.07019	6.191	[M+H]+1	trans-3-Indoleacrylic acid
C9 H8 O3	163.04024	12.422	[M-H]-1	Phenylpyruvic acid
C6 H5 N O2	124.03933	1.291	[M+H]+1	Nicotinic acid
C10 H7 N O3	190.0496	7.43	[M+H]+1	Kynurenic acid
				Others
C9 H6 O2	147.04378	7.618	[M+H]+1	2-Formylbenzofuran
C29 H50 O5	479.37264	35.95	[M+H]+1	Homocastasterone
C9 H6 O2	147.04378	11.112	[M+H]+1	Coumarin
C10 H16 O	153.12715	9.194	[M+H]+1	Citral
C9 H7 N O	146.05979	6.191	[M+H]+1	4-Indolecarbaldehyde
C15 H20 O4	265.14303	7.712	[M+H]+1	(±)-Abscisic acid
C11 H16 O2	181.12199	24.829	[M+H]+1	Dihydroactinidiolide
C8 H8 O	119.05041	12.424	[M-H]-1	Phenylacetaldehyde
C11 H14 O2	179.10641	14.241	[M+H]+1	(-)-Actinidiolide
C18 H36 O5	331.24932	24.457	[M-H]-1	Phloionolic acid

Formula	m/z	RT (min)	Adducts	Name
C6 H6 N2 O	123.05506	1.457	[M+H]+1	Nicotinamide
			Phenolic acids	
C7 H6 O5	169.01423	2.988	[M-H]-1	Gallic acid
C16 H16 O8	335.07762	12.539	[M-H]-1	5-Caffeoylshikimic acid
C9 H8 O3	165.0543	11.141	[M+H]+1	p-coumaric acid
C8 H8 O5	183.02989	15.333	[M-H]-1	4-O-Methylgallic acid
C7 H6 O3	139.03871	8.304	[M+H]+1	4-Hydroxybenzoic acid
C7 H6 O4	153.0195	5.128	[M-H]-1	Gentisic acid
C9 H8 O4	181.04917	7.955	[M+H]+1	Caffeic acid
C15 H12 O8	321.05998	8.325	[M+H]+1	Ampeloptin
C16 H18 O9	353.08755	9.013	[M-H]-1	Neochlorogenic acid
C14 H16 O10	343.06676	4.53	[M-H]-1	5-Galloyl quinic acid
C7 H6 O4	153.01939	4.626	[M-H]-1	3,5-Dihydroxybenzoic acid
C13 H14 O9	315.07053	9.484	[M+H]+1	1-Salicylate glucuronide
C14 H6 O8	300.99924	17.836	[M-H]-1	Ellagic acid
C14 H10 O9	321.02536	9.115	[M-H]-1	Digallic acid
C16 H18 O8	337.09315	7.305	[M-H]-1	3-P-coumaroylquinic acid
C16 H18 O9	353.08778	7.448	[M-H]-1	Chlorogenic acid
C13 H16 O10	331.06732	3.737	[M-H]-1	Glucogallin
C9 H8 O4	179.03471	6.252	[M-H]-1	Trans-Caffeic acid
C27 H24 O18	635.08974	12.895	[M-H]-1	1,2,6-Tri-O-galloyl-.beta.-D-glucopyranose
C25 H24 O12	515.12024	21.449	[M-H]-1	4,5-Dicaffeoylquinic acid
C7 H6 O3	137.02461	14.208	[M-H]-1	Salicylic acid
C16 H18 O9	353.08789	10.386	[M-H]-1	Isochlorogenic acid
C13 H16 O8	299.07736	6.97	[M-H]-1	2-(β -D-Glucopyranosyloxy)benzoic acid
C20 H20 O14	483.07818	9.726	[M-H]-1	2,6-Digalloylglucose
C7 H6 O3	137.02454	6.97	[M-H]-1	3-Hydroxybenzoic acid
			Phenols	
C6 H6 O3	125.02454	2.988	[M-H]-1	Pyrogallol
C7 H6 O2	123.04379	8.302	[M+H]+1	4-Hydroxybenzaldehyde
C7 H6 O3	139.03867	7.955	[M+H]+1	3,4-Dihydroxybenzaldehyde
C8 H6 O3	151.03866	11.878	[M+H]+1	7-Hydroxy-1(3H)-isobenzofuranone
C27 H22 O18	633.07327	9.487	[M-H]-1	Strictinin
C10 H12 O3	179.07137	7.878	[M-H]-1	Coniferyl alcohol
C9 H6 O3	163.03862	15.048	[M+H]+1	4-Hydroxycoumarin
C9 H6 O4	177.01944	11.319	[M-H]-1	5,7-Dihydroxycoumarin
C27 H24 O18	635.08904	14.722	[M-H]-1	2,4,6-Tri-O-galloyl-D-glucose
C20 H20 O14	483.07797	8.955	[M-H]-1	1,6-bis-O-galloyl-beta-D-glucose
C7 H7 N O2	138.05468	5.129	[M+H]+1	P-Hydroxybenzamide
C13 H16 O10	331.06713	2.678	[M-H]-1	6-O-Galloyl-glucose
C6 H6 O2	109.02967	5.128	[M-H]-1	Catechol
			Sugars	
C6 H12 O6	179.05622	0.787	[M-H]-1	D-Fructopyranose
C6 H12 O6	179.05618	0.907	[M-H]-1	Fructose
C7 H12 O8	223.04601	0.865	[M-H]-1	2-O-Carboxy-D-glucose
C6 H13 O9 P	259.02254	0.809	[M-H]-1	D-Glucose 6-phosphate
C18 H32 O16	503.16182	0.879	[M-H]-1	Glucosylsucrose
C6 H12 O6	179.05624	20.178	[M-H]-1	Fructofuranose

Formula	m/z	RT (min)	Adducts	Name
C12 H22 O11	341.10884	0.918	[M-H]-1	Sucrose
C6 H12 O6	179.05623	19.993	[M-H]-1	D-Mannopyranose
C6 H12 O6	179.05624	14.443	[M-H]-1	D-Galactopyranose
