# Supplementary figures and images for: Metabolomic Characterization of Sun-Dried Green Tea from Different Regions of Xishuangbanna
Source: Foods. 2026 Jul 15;15(14):2503. doi: 10.3390/foods15142503 (PMC13407954; doi:10.3390/foods15142503)

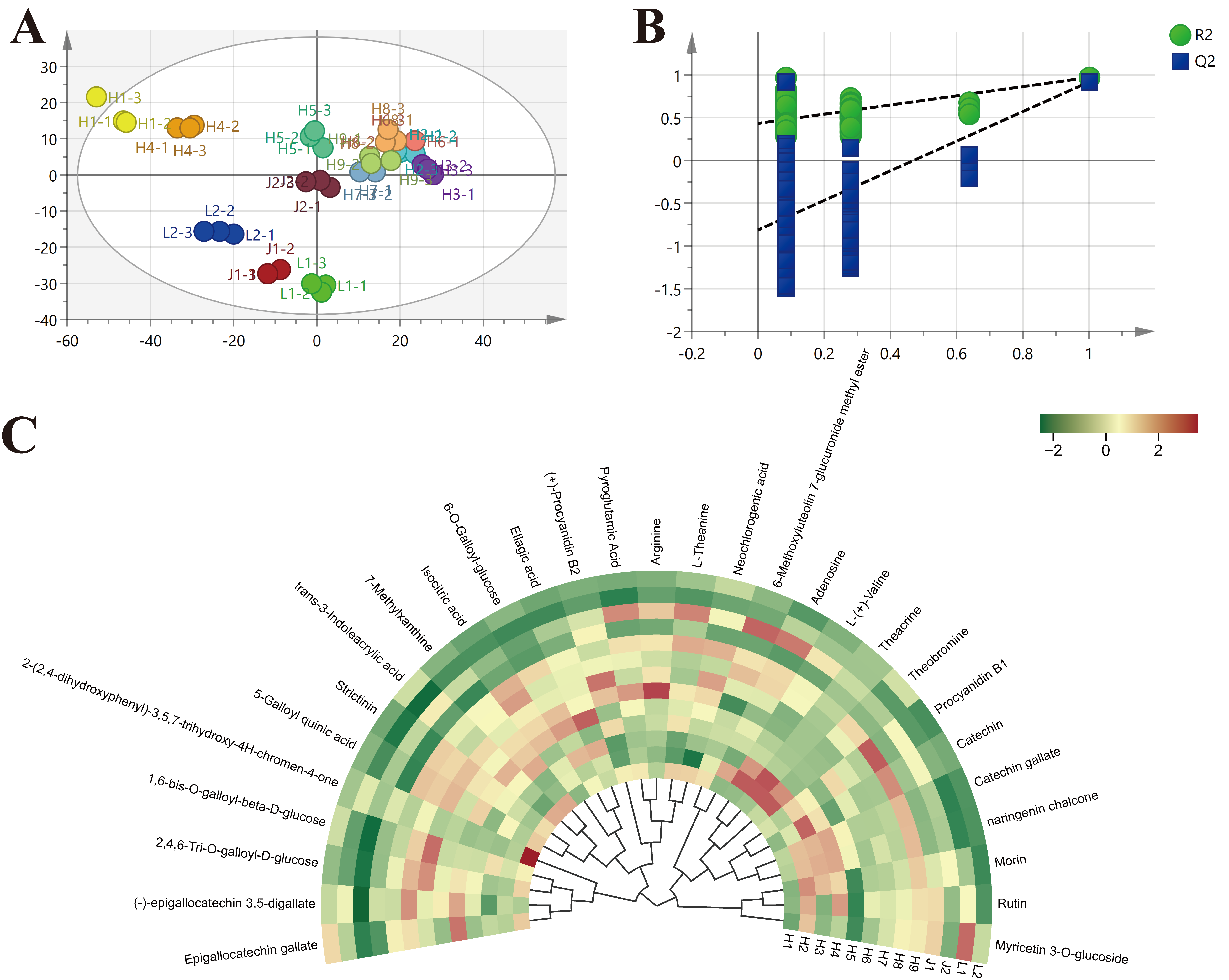

Supplement: Supplementary file 1 [file foods-15-02503-s001.zip › Figure S2.tif]
